# Exploring immunotherapy in colorectal cancer

**DOI:** 10.1186/s13045-022-01294-4

**Published:** 2022-07-16

**Authors:** Junyong Weng, Shanbao Li, Zhonglin Zhu, Qi Liu, Ruoxin Zhang, Yufei Yang, Xinxiang Li

**Affiliations:** 1 Department of Colorectal Surgery, Fudan University Shanghai Cancer Center, 270 Dong’an Road, Xuhui, Shanghai, 200032 China; 2 Department of General Surgery, Shanghai General Hospital, School of Medicine, Shanghai Jiao Tong University, Shanghai, 200080 China

**Keywords:** Metastatic colorectal cancer, Immune checkpoint blockade, Immunotherapy, Biomarkers, Immune desert, Microsatellite instability, Precision medicine

## Abstract

Chemotherapy combined with or without targeted therapy is the fundamental treatment for metastatic colorectal cancer (mCRC). Due to the adverse effects of chemotherapeutic drugs and the biological characteristics of the tumor cells, it is difficult to make breakthroughs in traditional strategies. The immune checkpoint blockades (ICB) therapy has made significant progress in the treatment of advanced malignant tumors, and patients who benefit from this therapy may obtain a long-lasting response. Unfortunately, immunotherapy is only effective in a limited number of patients with microsatellite instability—high (MSI-H), and segment initial responders can subsequently develop acquired resistance. From September 4, 2014, the first anti-PD-1/PD-L1 drug Pembrolizumab was approved by the FDA for the second-line treatment of advanced malignant melanoma. Subsequently, it was approved for mCRC second-line treatment in 2017. Immunotherapy has rapidly developed in the past 7 years. The in-depth research of the ICB treatment indicated that the mechanism of colorectal cancer immune-resistance has become gradually clear, and new predictive biomarkers are constantly emerging. Clinical trials examining the effect of immune checkpoints are actively carried out, in order to produce long-lasting effects for mCRC patients. This review summarizes the treatment strategies for mCRC patients, discusses the mechanism and application of ICB in mCRC treatment, outlines the potential markers of the ICB efficacy, lists the key results of the clinical trials, and collects the recent basic research results, in order to provide a theoretical basis and practical direction for immunotherapy strategies.

**Supplementary Information:**

The online version contains supplementary material available at 10.1186/s13045-022-01294-4.

## Introduction

Colorectal cancer currently ranks third in cancer incidence and second in cancer-related death globally. In 2020, there were 1,931,590 new cases of colorectal cancer worldwide and 935,173 deaths, accounting for 10.0% and 9.4% of the total number of cancer incidences and deaths, respectively [1]. In recent years, the research on the comprehensive treatment of colorectal cancer has continued to increase, and combination therapy with a targeted agent is the main treatment method for metastatic colorectal cancer (mCRC) patients [2, 3]. Whereas medication regimen is developing continually, it is difficult to make breakthroughs in mCRC therapy, prognosis remaining poor with a median overall survival (mOS) of only 25–30 months [4, 5]. Immunotherapy has achieved significant curative effects in the treatment of solid tumors, notably in melanoma and non-small cell lung cancer (NSCLC) [6, 7]. Immune checkpoint blockade (ICB) has enabled certain patients to obtain long-lasting benefits and have significantly improved disease prognosis. According to the latest follow-up data, the mOS of advanced melanoma was 72.1 months with Nivolumab plus Ipilimumab [8].These agents are used as the first-line treatment for patients with advanced solid tumor [9, 10]. However, inhibition of programmed death-1 (PD-1) or programmed cell death-ligand 1 (PD-L1) therapy has limited effects in the treatment of colorectal cancer. Until 2017, the first anti-PD-1 drug Pembrolizumab was approved by the Food and Drug Administration (FDA) as a second-line treatment for mCRC patients with microsatellite instability—high (MSI-H). However, there are only a few patients with dMMR/MSH-H (about 15% of colorectal cancer patients, 4% of patients with mCRC), and part of them enter into the stage of immune resistance soon [11–13].

Mutations in the DNA mismatch repair (MMR) genes can cause defects in the repair function during DNA replication, which leads to the occurrence of MSI. Both DNA deficient mismatch repair (dMMR) and MSI-H can lead to the accumulation of DNA mutations in tumor cells and results in generation of sufficient tumor neoantigens to enhance tumor immunogenicity, which can induce strong T cell and tumor immune response [14–16]. By contrast, mismatch repair-proficient (pMMR) colorectal tumor cells express weak immunogenicity and infiltrate a limited number immune cells, which makes it difficult to induce an adequate immune response [17, 18]. Therefore, ICB therapy is ineffective in such patients. In order to increase immunotherapy sensitivity, combined treatments are required to enhance tumor immunogenicity. With the development of research, the mechanism of immunotherapy resistance has been studied more thoroughly in mCRC. In addition to MSI, numerous biomarkers have been discovered and have been used to guide tumor immunotherapy, such as tumor mutation burden (TMB) [19, 20] and PD-L1 expression [21, 22]. However, due to the heterogeneity of the tumors, these markers are commonly used in other solid tumors and exhibit limited value in mCRC. It has been shown that MSS mCRC patients with POLE mutations can achieve an ideal immunotherapeutic effect [23]. More effective biomarkers are required for clinical use. The theory of tumor immunotherapy is complex, which is the result of multiple factors, temporal and spatial heterogeneity, and network co-regulation. A single theory or a single indicator cannot adequately explain the mechanism of immunotherapy. According to the genetic background, tumor microenvironment (TME), and the cell metabolism of mCRC patients, various models including CMS and CIRC subtypes have been proposed to guide the immunotherapy and comprehensive treatment of mCRC patients [24, 25].

Differences in the TME and in individual gene expression lead to diverse effects of immunotherapy. To date, a significant number of immunotherapy-related clinical trials have produced satisfying results which have led to the approval of Pembrolizumab and Nivolumab for mCRC treatment by the FDA [10, 26–30]. Other clinical trials, either ongoing or scheduled to initiate, are exploring the potential of activating inactive tumors [(“cold”) into (“hot”) tumors]. The clarification of the immune resistant mechanism is the premise to design innovative immunotherapeutic strategies. The present study focused on the following aspects: the current treatment opinions of mCRC, the mechanism of resistance ICB, the identification of potential biomarkers of the immune response, the key achievements of the latest clinical trials, and the breakthrough results of the preclinical studies. The analysis of this information aimed to demonstrate the application and treatment potential of the ICB therapy in mCRC.

## Treatment opinions of advanced colorectal cancer

**Fig. 1:**
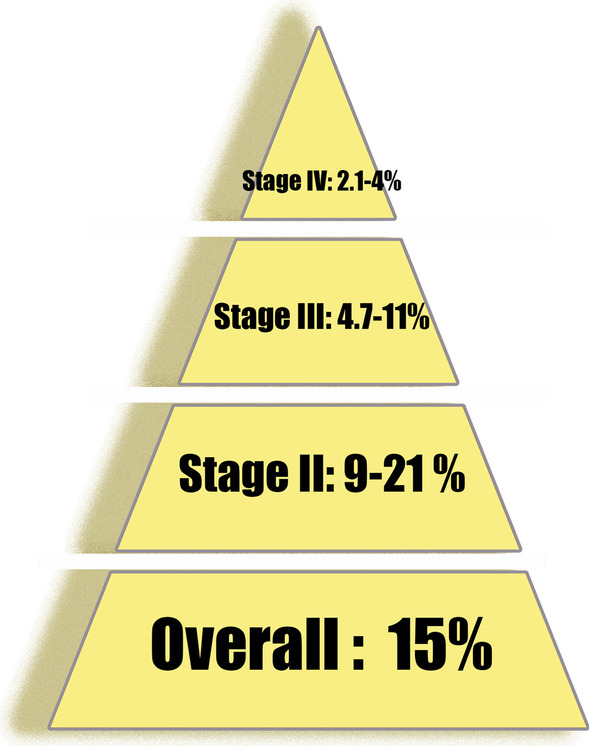
Proportion of dMMR/MSI-H in different tumor stages

Surgery is the primary treatment used for the majority of CRC patients. The majority of CRC patients with distant metastasis or recurrence do not receive radical resection. Surgery can only solve the tumor complications, such as intestinal obstruction, perforation, and bleeding, but it is not helpful for improving the survival of the patients. Comprehensive therapy, including chemotherapy, radiotherapy, immunotherapy, and targeted therapy, has become the main treatment opinion for these patients. Experts generally advise CRC patients to detect the mutations of KRAS and NRAS and guide targeted tumor therapy. They are also advised to assess the BRAF V600E status so as to stratify disease prognosis. In addition, MMR/MSI is recommended for all CRC patients to stratify prognosis and guide immunotherapy [2, 3]. Until the 2000s, 5-fluorouracil (5-FU) was the only alternative drug for advanced CRC, and the median survival time was no more than 1 year. In 1998 and 2002, irinotecan and oxaliplatin were approved by FDA to combine fluoropyrimidine for the treatment of advanced CRC, nearly doubling the survival. Then, combined with targeted drug (anti-VEGF, anti-EGFR or TKI), the median survival surpassed 2 years [5]. Regrettably, the large randomized phase III trial found that chemotherapy combined with two targeted drugs cannot further improve survival, but increase intolerable toxicity [31, 32]. Therefore, the traditional treatment of advanced CRC has entered the bottleneck again and is difficult to break through. Of note, Nivolumab and Pembrolizumab have been approved by the FDA for the treatment of mCRC [33, 34], due to their excellent performance [27]. ICB therapy is recommended for mCRC patients with dMMR/MSI-H, but immunotherapy resistance is observed in patients with pMMR/MSS [27]. According to previous studies, the proportion of dMMR/MSI-H decreases with the increase in the tumor stage. MSI is noted in approximately 9–21% of stage II and 4.7–11% of stage III tumors [35]. Its incidence is even lower in stage IV CRC (approximately 2.1–4%) [12, 36–38] (Fig. 1). Therefore, the application of immunotherapy in mCRC is very limited.

## The application of ICB therapy in CRC is limited

In Keynote-016, mCRC patients failed in prior standard treatment were divided into three cohorts based on their MMR status (MSI-H/dMMR CRC cohort, MSI-H/dMMR non-CRC cohort, and MSI-H/pMMR CRC cohort). Each cohort was provided with 10 mg/kg Pembrolizumab every 2 weeks, and the primary endpoint was objective response rate (ORR). The ORR was 40% (MSI-H/dMMR CRC cohort), 71% (MSI-H/dMMR non-CRC cohort), and 0% (MSI-H/pMMR CRC cohort), respectively [27]. It is deduced that mCRC patients with dMMR can benefit from PD-1/PD-L1 inhibitor, while the ICB therapy is ineffective in patients with pMMR.

**Fig. 2:**
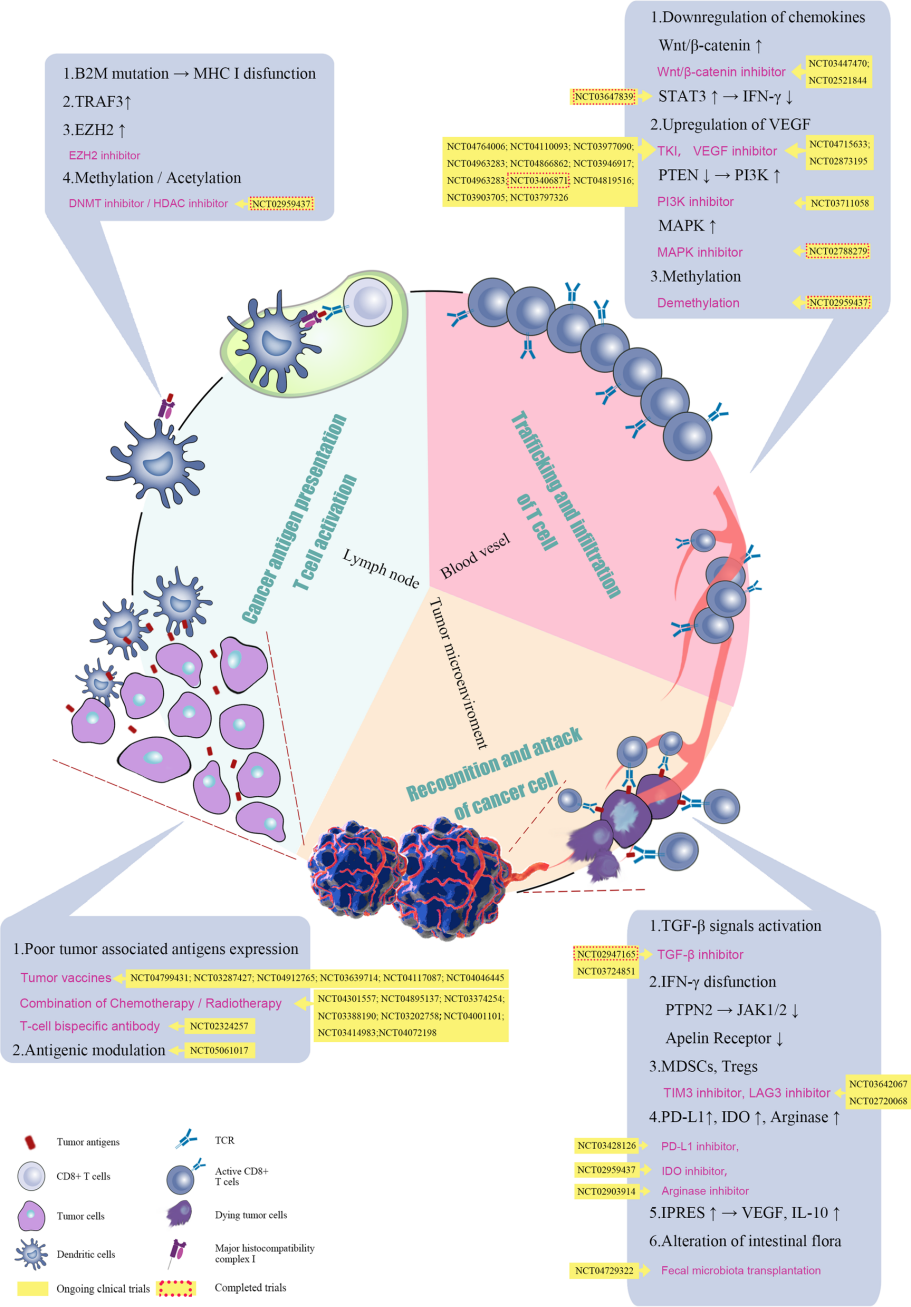
Mechanism of therapeutic resistance in ICB treatment. The reasons can be summarized as follows: insufficient tumor antigen presentation, tumor antigen presentation damage, T cell exclusion, and immunosuppressive signaling in the TME. The corresponding clinical trials are also marked in the figure

ICB therapy can activate the immune response of tumor lesions and repair the existing immune response by targeting the tumor-induced immune deficiency [39, 40]. It has been approved for the use in melanoma, NSCLC, head and neck squamous cell carcinoma and other malignant tumors and results in optimal clinical effects [22, 41–43]. Currently, the majority of mCRC patients are considered to be resistant to ICB therapy. The induction of tumor immune response involves omnifarious aspects, including tumor antigen presentation, T cell activation, T cell infiltration, and T cell recognition, which ultimately activate tumor cell killing. Any defect in these processes can lead to primary or acquired immune resistance [44]. The resistance mechanism of immunotherapy is extremely complex and is related to genetic factors and previous treatment of the patients. It is generally believed that immunotherapy resistance of CRC may be related to the following reasons: insufficient tumor antigen presentation, tumor antigen presentation damage, T cell exclusion, and immunosuppressive signaling in the TME (summarized in Fig. 2).

### Absence or loss of tumor antigens

Neoantigen is a new antigen encoded by mutated genes of tumor cells, which is an abnormal peptide mainly generated by gene point mutations, deletion mutations, and gene fusions. Its structure is different from that expressed by normal cells [45, 46]. The tumor antigen is the target of the immune system and aims to recognize cancer cells. It is the starting step of the antitumor immune response, which is notably important for the antitumor effect of ICB. Tumor-associated antigens (TAAs) are specific proteins that are overexpressed in tumor cells. They are also expressed in normal cells and can be detected by immune cells in order to trigger an immune response [47]. TAAs limit the ability of immune cells to recognize and induce relative weak specific immune response. CEA is an important TAA of CRC and is frequently expressed on the surface of the majority of mCRC cells. It induces immune tolerance owing to its occurrence in the embryonic stage [48]. To address this disadvantage, the T cell bispecific antibody (TCB) has been employed to strengthen T cell engagement in TME. CEA-TCB induces an interaction of cancer cells with T cells via binding to CEA and CD3. In a phase I trial (NCT02650713), combination of CEA-TCB with ICB in CEA-positive solid tumors indicated a partial response (PR) of 20% (MSS status) and a stable disease (SD) of 50% [49]. In addition, under the pressure of antitumor immunity, the tumor antigens are reduced or lost. This process is termed antigenic modulation and enables tumor cells to escape immune recognition and killing. There is insufficient mutant load to express tumor antigens that produce focused CD8^+^ T cell responses. These tumors do not respond to ICB therapy due to a lack of T cells specific for distinct tumor antigens [46]. In fact, tumors with a high mutational load and overexpression of tumor neoantigens, such as melanoma, head and neck, NSCLC and MSI tumors, are generally more sensitive to ICB therapy. The majority of the CRC patients exhibited a relatively low mutational load. In order to solve the problem of tumor antigen deficiency or defect, tumor vaccines have become a major focus of investigation. The purpose is to introduce tumor antigens (including tumor cells, tumor-related proteins or peptides, and genes expressing tumor antigens) into patients, enhance immunogenicity, activate the patient’s own immune system, and induce the body’s immune response, so as to control or eliminate the tumors [50]. In April 2010, the FDA approved the cancer vaccine PROVENGE® for the treatment of metastatic castration-resistant prostate cancer. PROVENGE® became the first and, to date, the only therapeutic cancer vaccine [51]. From October 20, 2021, 2,119 tumor vaccine-related clinical trials have been registered on the *clinicaltrials.gov* Web site. However, the majority of the phase III clinical trials ended in failure due to nonsignificant improvement in the OS of the patients [52]. However, the development of tumor vaccines is still one of the major breakthroughs to improve immunotherapy, along the whole omics development path, gradually from cell to protein and then specific to gene, and now it has entered into the stage of nucleic acid vaccine. It is also a transition from a general tumor vaccine to a tumor personalized vaccine for precise treatment, as well as the transition from TAA to tumor-specific antigens (TSAs) [53].

### Tumor antigen presentation damage

Tumor antigens are displayed on the cell surface through major histocompatibility complex class I (MHC-I) molecules. Lack of antigen presentation causes tumor cells to induce tolerance toward T cells, which includes the two following parts: a. Tumor antigen is absorbed by dendritic cells (DCs) and cross-presented to initiate CD8^+^ T cell activation; b. the antigen is directly presented by the tumor cells so that the activated CD8^+^ T cells can recognize and kill them [54]. Tumor cells can use diverse escape mechanisms to evade the immune recognition from these two steps. Lost or low expression of MHC-I molecules has been reported on the surface of tumor cells, which results in the obstacle of tumor antigen presentation and the inability to provide the first signal for T cell activation [55]. Previous studies have shown that an antigen-specific T cell level close to 1% is only likely to initiate an effective antitumor response. However, current studies have shown that the antigen presentation level of the majority of cancer cells is very low or even absent, resulting in a weak immune response.

Various mechanisms have been proposed that disrupt antigen presentation in CRC, including interference with the process of proteasome processing of antigens, regulation of the function of transporter associated with antigen processing (TAPs), and obstruction of the expression of MHC structural components through gene mutations, which are notably found in MSI-H tumors [56, 57]. CRC patients with TAPs and MHC-I positive expression were accompanied by increased infiltration of CTLs, leading to subsequent tumor response [58]. β-2-microglobulin (β2M) plays a role in MHC transportation and stable expression on the cell surface. The loss of heterozygosity of β2M can affect the antigen presentation of MHC-I, which leads to melanoma resistance to T cell infiltration and induces primary and acquired ICB resistance [59, 60]. A MSI-H mCRC patient who possessed typical MSI-H molecular characteristics including high mutation load demonstrated disease progression during ICB therapy. Dung et al. surprisingly found that this patient had a loss of β2M biallelic genes, which may be an important reason for his primary resistance to ICB treatment [10]. In addition, the EZH2 inhibitor can overcome ICB treatment resistance by reducing the histone H3K27me3 modification on the β2M promoter [61, 62]. Methylation and histone acetylation can significantly affect the antigen processing and surface presentation of MHC. In lymphoma and melanoma models, both demethylating agents and histone deacetylating agents can increase MHC expression, resulting in increased infiltration of CD8^+^ T cells and subsequent induction of the antitumor response [63, 64].

### T lymphocyte exclusion

T cells are the central link of the immune response, and the lack of T lymphocytes in the TME is a direct and fundamental cause of immunotherapy failure. The lack of tumor antigen or impairment of tumor antigen presentation described above can both affect the recognition of tumor cells by CD 8^+^ T lymphocytes and indirectly affect T lymphocytes infiltration into TME. Of note, a deficiency in T lymphocytes has been noted in the TME, which is termed T lymphocyte exclusion [65]. Differential expression of chemokine receptors is required for efficient T cell homing and recruitment in the TME [66]. In particular, CXCR3 has been identified as a chemokine receptor critical for T cell infiltration [67]. It has been shown that the Wnt/β-catenin signaling is frequently activated and associated with T cell exclusion in CRC, which is the major obstacle for immunotherapy [68]. Previous studies have shown that activation of the Wnt pathway and expression of nuclear β-catenin are inversely correlated with the infiltration of T cells in CRC tissues. ICRT14 is an inhibitor of β-catenin/T cell factor (TCF), which potently enhances T cell and natural killer cell (NK cell) infiltration. The expression of chemokine (C-X-C motif) ligand 9/10/11 (CXCL9/10/11) was inhibited by activation of the Wnt/β-catenin signaling, suggesting that suppression of β-catenin is expected to shift the colorectal cancer microenvironment into a T cell inflammatory phenotype and enhance the efficacy of immunotherapy [69–71]. Clinically, melanoma tumors with Wnt/β-catenin activation respond poorly to ICB, whereas they produce a strong response without Wnt/β-catenin mutations. Inhibitors of the Wnt/β-catenin pathway are intensively investigated in clinical trials and can be combined with ICB to overcome this pattern of primary resistance [72].

Furthermore, signal transducer and activator of transcription 3 (STAT3) can decrease the ability of CD8^+^ T cells to produce interferon in tumors-γ (IFN-γ). This in turn inhibits CXCL10 secretion by tumor-associated myeloid cells and prevents T cell recruitment. The mitogen-activated protein kinase (MAPK) signaling cascades upregulate the expression levels of the immunosuppressive cytokines vascular endothelial growth factor (VEGF) and interleukin 8 (IL-8), which inhibit T cell function and recruitment into the tumors [73, 74]. Inhibition of the MAPK cascade improves CD8^+^ T cell infiltration and may also sensitize tumors to ICB therapy [75]. This provides a strong rationale for combination therapy of multi-kinase inhibition and ICB. Similarly, loss of phosphatase and tensin homologue (PTEN) leads to activation of phosphatidylinositol 3-kinase (PI3K) signaling, associated with an increase in the expression of VEGF and reduction in CD8^+^ T cell infiltration [75]. Epigenetic alterations including DNA methylation and histone modifications have also been considered as an important mechanism of chemokine inhibition and tumor progression. Therefore, treatment with epigenetic modulators can increase chemokine expression and T cell infiltration in the TME.

### T cell suppression in TME

CRC development is a complex multifactorial process, during which CRC cells and their surroundings constitute a specific TME. Cancer cells interact and co-evolve with TME, thereby promoting tumor initiation and progression. Several factors in the TME act to suppress immune function, including regulatory T cells (Tregs), IL-10, tumor-associated macrophages (TAMs), myeloid-derived suppressor cells (MDSCs), and related cytokines, which can affect ICB therapy and lead to drug resistance [76].

Previous studies have shown that resistance in ICB therapy can occur even with CD8^+^ T cell infiltration in the TME, which may be due to the lack of the IFN- γ response. It is known that, during antigen-specific immunity, IFN-γ is mainly secreted by CD8^+^ cytotoxic T cells and by CD4 Th1. The IFN- γ binding with the IFN-γ receptor (IFNGR) leads to Janus kinase 1 (JAK1) and Janus kinase 2 (JAK2) activation and subsequent recruitment and phosphorylation of STAT1 [77]. This complex translocates to the nucleus, where it activates interferon regulatory factor 1 (IRF1). The transcriptional activity of this factor ultimately leads to an IFN-γ-mediated antitumor effect, as well as increased PD-L1 expression [78–81]. Tumors that have a high mutational load are more likely to respond to ICB therapy. However, certain patients who do not respond despite a high mutational load can present with JAK1/JAK2 mutations. Similarly, functional loss of JAK1/JAK 2 mutations has been found in melanoma samples and melanoma cell lines, which fail to respond to IFN-γ signaling and result in lack of PD-L1 expression. Moreover, a CRISPR screen assay found that apelin receptor interacted with JAK1 and regulated IFN-γ response [82]. It was further suggested that activating mutations were present in protein tyrosine phosphatase non-receptor type 2 (PTPN2), which negatively regulated JAK1 and STAT1 signaling. These were both associated with resistance to ICB therapy caused by the reduced response to IFN-γ. Conversely, CRISPR-Cas9 genome editing can restore melanoma resistance to IFN-γ sensitivity owing to PTPN2 loss [83]. PD-L1 expression may reflect the IFN-γ response, and consequently PD-L1 expression can predict to some extent the clinical efficacy of ICB therapy. However, genetic mutations in IFN-γ signaling genes are uncommon in CRC patients and occur in less than 10% of patients with colorectal adenocarcinoma [84]. Loss of function alteration including JAK1 frameshift is noted in lower than 3% in MSS colon adenocarcinoma samples [85].

Therapy resistance of ICB occurs despite adequate CD8^+^ T cell infiltration and IFN-γ response when some certain non-tumor cells (NTCs) existed in TME. Tregs and MDSCs with the ability to modulate local immune functions are considered the representative of NTCs. When Tregs or MDSCs are present in the TME, they lead to a reduced immune response against tumor cells. Several studies have shown that depletion of MDSCs or Tregs in the TME can reverse ICB therapy resistance [86–88]. Tumor cells and their surrounding stroma can co-regulate the immunosuppressive microenvironment, which leads to resistance to ICB therapy. Therefore, journal of *Cell* has profiled the transcriptomic and genomic features of metastatic melanoma patients during their treatment with ICB [89]. During the enrichment of BRCA2 mutations, high expression levels of the DNA repair genes were observed in the responding patients. By contrast, tumors with the innate PD-1 resistance (IPRES) signature demonstrate upregulation of genes involved in the regulation of multiple biological processes, including local immunosuppressive genes (VEGF, IL-10), and genes involved in monocyte/macrophage/MDSC chemotaxis, angiogenesis, mesenchymal transition, and wound healing. It is important to note that MAPK-targeted therapies can induce a similar emergence of transcriptional signatures in melanoma, implying that mitogen-activated protein kinase kinase (MEK) inhibitor therapies may be cross-resistant with ICB therapies. IPRES signature is a transcriptomic representative of TME existing in various cancer types, including melanoma, renal clear cell carcinoma, and colon adenocarcinoma. The relevance between this pattern with CRC may provide a unique method to predict the ICB response. Interestingly, the intestinal flora microenvironment is associated with ICB treatment. There are more than 100 trillion bacteria in the human intestine, which form a complex microflora microenvironment that can regulate metabolism and immune function. Since 2011, one after another researches reported that the intestine microflora can function in immune modulation. In 2015, two articles were simultaneously published in the journal *Science* to discuss the use of ICB in melanoma [90, 91]. Two distinct groups reached the following similar conclusions: Antibiotics can disrupt the antitumor effects of ICB. Specifically, antibiotic-treated mice that were administered with deficient intestinal microflora could restore the ICB’ anticancer effects; patients with specific microbiota, such as Bacteroides and Bifidobacterium, exhibited improved outcomes in immunotherapy. This is the first time that the intestinal microflora was linked with the efficacy of ICB. Subsequently, significant research findings were published validating this theory. Routy et al. demonstrated that *Akkermansia muciniphila* existed in the majority of patients who were treated with ICB and could achieve remission [92]. Gopalakrishnan et al. demonstrated that the profile of intestinal microflora was associated with the efficacy of ICB therapy. Their study showed that the 30 patients who responded to ICB treatment exhibited a significantly different microflora from the 13 patients who did not respond, and the two specific bacteria, *faecalibacterium* and *clostridiales*, were prevalent in progression-free survival (PFS) patients [93, 94]. Recently, a phase I clinical trial (NCT03353402) examining the fecal microbiota transplantation (FMT) was performed in 10 ICB refractory metastatic melanoma patients. Two patients with a partial response (PR) and one patient with a complete response (CR, near complete disappearance of tumor cells) were observed. PFS crossed the 6-month milestone in all responders [95]. For mCRC, a clinical trial in MSS patients demonstrated that patients with high abundance of *fusobacterium* had significantly shorter PFS (median PFS = 2.0 versus 5.2 months; *p* = 0.002) [96]. A recent study indicated that *fusobacterium* was present in both primary tumors and liver metastases in mCRC. Its presence was significantly associated with poor prognosis [97]. Viaud et al. suggested that the metabolic functions of bacteria present in tumor cells may be responsible for the immune resistance of mice with colon cancer [98]. The aforementioned studies indicated that the disturbance of the intestinal flora microenvironment could promote the immune escape of tumor cells, leading to immunotherapy resistance. Therefore, the gut microbiota may be one potential factor influencing the response to immunotherapy in CRC.

## Predictive biomarkers of response in ICB therapy

**Fig. 3:**
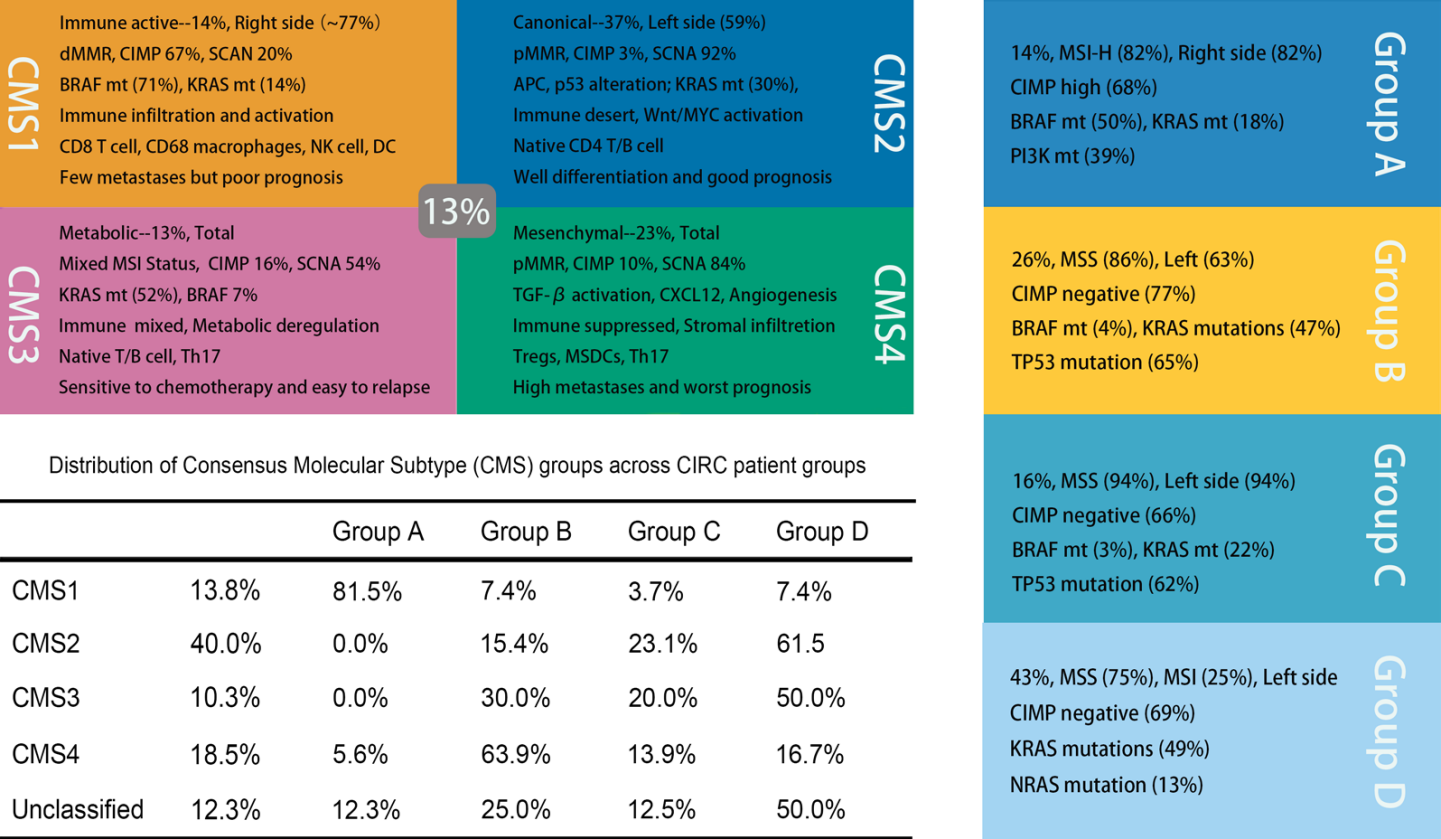
Consensus molecular classification (CMS) and the coordinate immune response cluster (CIRC) typing of CRC

The reduced efficacy of ICB therapy in mCRC patients led to the subsequent investigation of this pathway and its contribution in the response to cancer therapy. Colorectal tumorigenesis is a multistage, multistep, and multigenetic process, with high degrees of genetic heterogeneity. During CRC progression, different key genes and different signaling pathways act at different stages. The current consensus molecular classification (CMS) of CRC approved by the academic community consists of the following five types (Fig. 3): CMS1 is an immune-activated type with MSI-H, which presents with mutations in mismatch repair genes and accounts for approximately 14%. CMS2 is a classical type with aberrant activation of the Wnt and Myc signaling pathways, which harbors significant somatic copy number variation and accounts for 37% of cases. CMS3 is a metabotropic type with a high rate of KRAS mutations, accounting for approximately 13%. CMS4 is the mesenchymal type with abnormal activation of the transforming growth factor β (TGF- β) signaling pathway, noted in approximately 23% of cases. Finally, 13% of the cases cannot be classified alone into any of the aforementioned categories and are classified as mixed type [24].

The majority of the CMS1 tumors are located in the right colon, with deep local invasion and poor differentiation, while a limited number of cases develop distant metastasis. CMS1 has a low frequency of KRAS mutations, whereas BRAF is mutated at a high frequency. Its OS and PFS are poor. BRAF mutations represent poor responsiveness to standard chemotherapy and EGFR targeted therapy, and they are considered as a major reason for the poor prognosis of patients with this type. Different CMS subtypes of CRC exhibit different characteristics in their TME, which is important for developing treatment strategies. CMS1 TME exhibits abundant infiltration of CD8^+^ lymphocytes, CD68^+^ macrophages, and adequate expression of PD-1 and PD-L1. Optimal clinical outcomes can be achieved from ICB therapy. CMS4 is associated with an “immunosuppressive phenotype” and is presented in immune tolerance. CMS4 is infiltrated by Tregs, MDSCs, monocyte-derived cells, and T helper 17 (Th17) cells and is associated with high expression of CXCL12 and TGF-β. In this type of cells, TGF-β and angiogenesis-related factors play important roles in immune evasion. Preclinical and clinical studies have demonstrated that TGF-β inhibitors can reverse immune tolerance into immune sensitization [99]. The CMS2 and CMS3 CRC cells are described as “immune desert” and “cold” tumors, which are noted during primary resistance to immunotherapy [100]. Based on this evidence, different types of CMS patients require continued in-depth exploration of their mechanisms of immune tolerance in the search for more effective immunotherapeutic strategies [101–103].

Another molecular type used to describe the immunotherapy response in a population of CRC is the coordinate immune response cluster (CIRC) typing (Fig. 3), which primarily divides CRC patients into four groups, including groups A, B, C, and D, based on cluster expression of a gene set (Table 1) [25]. Group A patients exhibits high expression of CIRC genes and were characterized by MSI-H, and upregulation of several immune checkpoint genes, including CTLA4, PD-L1, LAG-3, and TIM3. In contrast to these observations, hypermutations in KRAS, BRAF, NRAS, TP53, PIK3CA, and PTEN, have been reported in the group of lower CIRC expression. This links the genetics and immunobiology of CRC carcinogenesis, providing a theoretical and practical basis for immune stratified therapy. The frequent mutations of MSI-H and POLE mutations were observed in Group A and were associated with high mutational load and high immune infiltration, which suggests that patients can benefit from ICB therapy. Although group D patients enriched in RAS mutations are resistant to ICB treatment, novel strategies have to be developed to address potential resistance in this patient population.

**Table 1:** Genes within the coordinate immune response cluster

Gene ID						
HLA-DQA1	HLA-DQA2	HLA-DRB5	HLA-DMA	PDCD1LG2	ICAM1	CD274
STAT1	IRF1	IFNG	CTLA4	TBX21	CCL5	LAG-3
CD247	ICOS	IL18RAP	GNLY	CXCL10	HLA-DPB1	HLA-DPA1
HLA-DMB	HLA-DRA	HLA-DMA	CD80	HLA-DOA	CD4	HAVCR2

The aforementioned molecular typing is of great significance for the treatment of CRC patients. Recently, a clinical trial based on the CMS principle is ready to be performed for the assessment of the clinical application and prediction value [104, 105]. It is interesting to note that CMS1 and Group A are both immune-activated and respond well to ICB therapy. Moreover, BRAF mutation is frequently accompanied by MSI-H in these two types of CRCs [106]. BRAF mutation was found to be a negative prognostic marker in CRC, and it could promote the development of TME [107, 108]. Therefore, it was speculated that BRAF mutations may possess predictive value in immunotherapy. A meta-analysis was performed comparing the ORR of immunotherapy in MSI-H CRC patients with BRAF mutant and wild-type patients. The results indicated no significant differences in ORR in response to immunotherapy between BRAF mutant and wild-type patients. The results suggested that BRAF mutation did not exhibit predictive value in MSI-H mCRC immunotherapy [109]. However, immunotherapy, as an important complementary method, is still ineffective in the treatment of the majority of CRC patients. Significant investigations have been performed to address the ability of specific indicators to predict the efficacy of ICB therapy.

### Microsatellite instability

The MMR genes encode the corresponding mismatch repair protein. Four principal genes are associated with genomic instability as follows: MLH1, MSH2, MSH6, and PMS2 [110]. Defects in DNA mismatch repair system can cause MSI [111]. Generally, the MSI-assay evaluates five selected microsatellite loci as follows: Bat-25, Bat-26, d5s346, d2s123, and d17s250. The MSI status was classified as high instability (MSI-H), low instability (MSI-L), and stability (MSS) [112]; MMR is divided into deficient mismatch repair and proficient mismatch repair. Clinically, dMMR is equivalent to MSI-H, pMMR to MSI-L or MSS [113]. Accumulation of mutated and unrepaired genes will cause MSI-H and induction of carcinogenesis. Approximately 15% of CRCs are caused due to activation of the MSI pathway [38, 111, 114, 115]. MSI-H is associated with abundant neoantigen production, which causes higher immunogenicity and potent immune responses [27]. In CRC, MSI is an excellent predictive biomarker, and as a result, the FDA has approved Pembrolizumab in the treatment of MSI-H/dMMR mCRC patients [116].

### Tumor mutational burden

TMB is the number of total mutations per megabase of tumor cells in the coding region, which is used as another predictor of ICB treatment efficacy [117]. The positive correlation between TMB and the efficacy of ICB in immunotherapy was reported for the first time in 2014 [118]. The KEYNOTE-158 study demonstrated that high TMB was associated with better OS, and that TMB could be used as a potential pan-cancer biomarker [19]. In a prospective planned retrospective analysis of various solid tumor patients treated with Pembrolizumab, the ORR was 29% in the TMB high (TMB-H) group compared with only 6% noted in the non-TMB-H group. However, colorectal cancer was not included [19]. Based on this evidence, FDA-approved Pembrolizumab on June 16, 2020 for the treatment of refractory unresectable or metastatic solid tumors of TMB-H, defined by 10 mutations/megabase (muts/MB) based on the FoundationOne CDx assay. However, a latest multicenter, open-label, phase 2a multiple basket study in pan-cancers [119], which accounts for the majority of CRC, reported the value of TMB as a predictor of atezolizumab treatment. The results exhibited that the ORR of MSI-H and MSI-L in patients with TMB ≥ 16 mut/MB was 54.5% and 31.0%, respectively. The median PFS (mPFS) was 8.3 months and 5.6 months, respectively; and the median OS was NE and 19.8 months, respectively. In MSI-L population, the PFS of patients with TMB ≥ 16mut/MB was significantly better than that of patients with 10 ≤ TMB < 16 mut/MB (HR = 0.33, *P* < 0.0001)). As shown from the data of Aaron et al., MSS/TMB-H accounted for 5.36% (7, 972/148, 803) of cancer cases, while MSI-H only accounted for 1.46% (2, 179/148, 803); thus, it was initially speculated that the use of TMB could make more potential mCRC patients benefit from immunotherapy [120]. David et al. also found a similar phenomenon in patients with MSS [121]. Moreover, studies also found that TMB could be used as an independent biomarker for ORR, PFS, and OS in patients with MSI-H mCRC [122]. In general, the detection of TMB requires tumor specimens for sequencing, termed tTMB, while advanced-stage patients do not have conditional access to tissue samples. Therefore, various clinical centers have attempted to use blood samples to detect bTMB. Certain clinical trials demonstrated that bTMB was significantly associated with tTMB [123–126]. In a clinical trial of Durvalumab in combination with Tremelimumab in refractory mCRC, it was found that the MSS mCRC patients with bTMB ≥ 28 muts/MB benefitted from ICB treatment [127]. This evidence suggests that bTMB may be an efficacy predictor in the MSS mCRC population, although the specific cutoff value required further exploration and validation.

It is interesting to note that TMB metrics are also flawed, since certain patients with high TMB exist who do not respond to immunotherapy [128] or patients with low TMB who can also achieve optimal therapeutic responses [129–131]. This is due to the fact that a high TMB does not correspond to a high tumor neoantigen level. It is therefore clear that the mutation quality is much more important than the mutation quantity. However, tumor neoantigen burden (TNB) is an indicator that reflects the total neoantigen quantity in tumor cells, which can be used as an adjunct to the TMB indicator [132].

### Expression levels of PD-L1

The predictive role of PD-L1 in the immunotherapy of solid tumors has been affirmed by various studies. It is generally believed that high expression of PD-L1 is associated with sufficient immune response and clinical benefits from ICB treatment [133]. However, the conclusions from multiple trials were not consistent. An additional study indicated no significant difference in the immune response rate between the PD-L1-positive subgroup (≥ 1%) and the negative subgroup (≤ 1%) [134]. Some researchers believe that PD-L1 expressing should be distinguished from different cells. In a 73 MSI CRC patients cohort, Overman et al. evaluated the relationship between the efficacy of Nivolumab and the expression of PD-L1 on tumor cells or immune cells. The results showed that there was no significant correlation between PD-L1 expression on tumor cells and immunotherapy response, but it was found that ORR with numerous expressions on immune cells was significantly improved [29]. Le et al. reported that PD-L1 expression was only observed in MSI patients, while it was previously speculated that MSS tumors with high PD-L1 expression may also respond to ICB treatment [27, 135]. Subsequently, 138 CRC patients were recruited in O'Neil's study to detect the expression levels of PD-L1. PD-L1-positive CRC patients received Pembrolizumab treatment, and only 4% (1/23) obtained PR. This was also noted in the MSI status [136]. Obviously, the real challenge of immunotherapy is to find biomarkers of MSS CRC, but the current research on PD-L1 has not broken through the dilemma [137]. The reason for this discrepancy among different clinical trials may be that immunohistochemistry was the most commonly used method to detect PD-L1. However, certain differences have been noted with regard to the immunohistochemical antibodies and the scoring systems adopted by different centers, which leads to the incompatibility of the results. The use of different antibodies resulted in significant differences in the evaluation of results [138]. In upper gastrointestinal cancer, the score of PD-L1 has been standardized, and the Combined Positive Score (CPS) > 1 is the critical value of pembrolizumab immune response [139]. It is worth mentioning that the standardized antibody (22C3 pharmDx IHC assay) is used in CPS [139]. Therefore, a unified standard should be formed for CRC so that the most suitable critical value can be determined. In addition, the uneven distribution of PD-L1 in tumors and stromal cells leads to the inconsistency between biopsy specimens and resected tissues [140]. Therefore, the lesions cannot be completely removed, and simple biopsy increases the probability of false negative results. Compared with single-core biopsy, multi-core biopsy is more sensitive to the detection of PD-L1 [140]. The expression levels of PD-L1 were significantly altered in the initial stage of the disease, during its progression and following treatment. Kelly et al. demonstrated that 50% of patients with advanced esophageal adenocarcinoma exhibited altered PD-L1 status (from negative to positive PD-L1 expression) following radiotherapy and chemotherapy [141]. In conclusion, additional prospective studies using unified standards to examine the tumor therapeutic response of CRC patients are required to explore and validate the efficacy of PD-L1.

### IFN-γ

In the TME of CRC, tumor-infiltrating CD8^+^ T cells, NK cells, and NK T cells are the main producers of IFN-γ. IFN-γ can in turn prompt more CD8^+^ T cells and NK cells infiltration into TME [142]. IFN-γ promotes the MHC-I expression both in antigen-presenting cells (APCs) and in tumor cells, enhancing the antigen recognition function of CD 8^+^ T cells to kill tumor cells. Furthermore, IFN-γ boosts Th1 cells polarization and directly induces tumor cells apoptosis or non-apoptotic death [143, 144]. IFN-γ can also establish tumor cells dormancy and inhibit tumor cells proliferation by IFN-γ/STAT1 pathway [145] or non-STAT1 signaling [146]. Nevertheless, IFN-γ may also promote tumor immune evasion by promoting tumor cell dormancy under certain condition. IFN-γ can induce tumor antigen loss, recruit MDSCs and TAMs into the TME, and induce tumor immunoediting which results in tumor progression and relapse [147]. In addition, IFN-γ facilitates the expression of immunosuppressive molecules PD-L1, indoleamine 2,3-dioxygenase (IDO), and arginase in TME. Clinically, a relatively high level of IFN-γ was associated with response of immunotherapy [148, 149]. However, shortage or excess of IFN-γ signaling may also lead to immune resistance [81, 150].

**Fig. 4:**
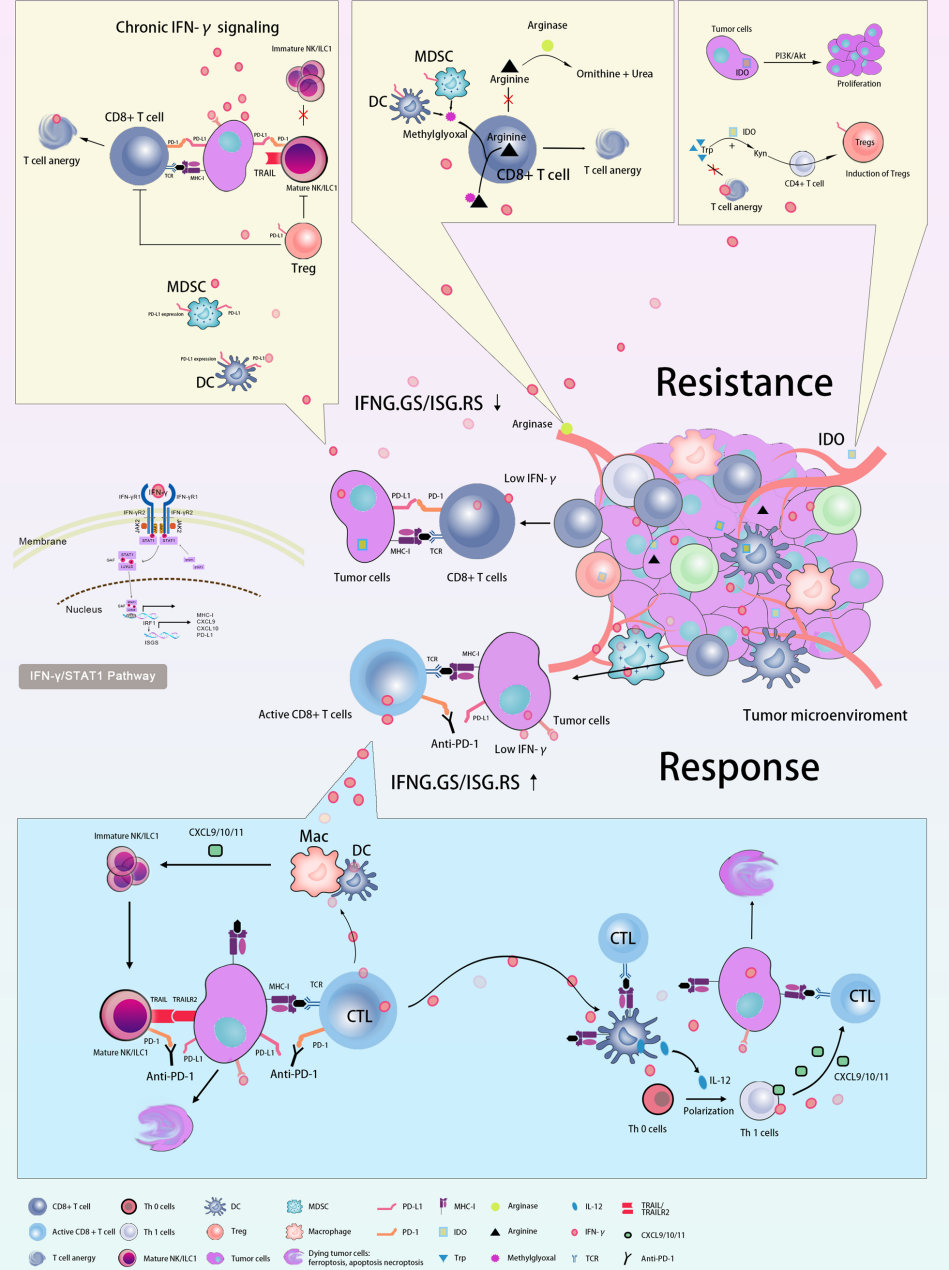
Controversial roles of IFN-γ in the TME of CRC. Antitumor: IFN-γ signaling from immune cells and a high IFNG.GS/ISG.RS ratio were associated with increased CD8+ T cells and NK cell activation, and high response to ICB immunotherapy. IFN-γ can prompt CD8+ T cells and NK cells infiltration into TME, promote the MHC-I expression, boost Th1 cells polarization, directly induce tumor cells apoptosis or non-apoptotic death, establish tumor cells dormancy and inhibit tumor cells proliferation by IFN-γ/STAT1 pathway or non-STAT1 signaling; immune evasion: IFN-γ signaling released by tumor cells and a low IFNG.GS/ISG.RS ratio were associated with resistance to ICB treatment. IFN-γ can induce tumor antigen loss, recruit MDSC and TAMs into the TME, induce tumor immunoediting, and facilitate the expression of PD-L1, IDO, and arginase in TME

Therefore, the role of IFN-γ in the TME is controversial (summarized in Fig. 4). Recently, Joseph et al. analyzed single-cell sequencing data of IFN-stimulated genes (ISGS) [151] in various cell populations in melanoma samples from the TCGA repository and the data demonstrated that the ISGS resistance signature-related genes (ISGS. RS) were mainly expressed in cancer cells. By contrast, IFNG.GS was predominantly expressed by immune cells within the TME, such as T cells, NK cells, and macrophages. Interestingly, a low IFNG.GS/ISG.RS ratio was associated with resistance to ICB treatment. However, a high IFNG.GS/ISG.RS ratio was associated with increased CD8^+^ T cell and NK cell activation, and high response to ICB immunotherapy. Particularly, the immunotherapeutic response predicted by this ratio was independent of TMB. Previous studies have shown that IFN-γ signaling released by tumor cells can limit the immune response. However, IFN-γ signaling from adaptive and innate immune cells can enhance immune responses. These two actions of the same process are completely opposite. This intriguing study may provide us with an explanation for the dual role of IFN-γ in tumor immunity. It also highlights the potential of administrating therapeutic strategies for patients with different IFN-γ status.

### Tumor-infiltrating lymphocytes (TILs)

TIL is an important component of the TME, and TILs have been associated with upregulation of PD-L1 expression and various clinical benefits. The antitumor effects of ICB require the participation of lymphocytes in the vicinity of the tumor. Therefore, the abundance of TILs can also be used as a marker to predict the efficacy of the ICB. Normally, immunohistochemical analysis is used to assess the infiltration of CD8^+^ T cells in the tumor tissues. As a co-stimulatory signal, a higher proportion of CD28^+^ in TIL cells generally predicts a higher response to therapy, which can also be detected by immunohistochemical analysis [152]. It was reported that only small fractions of the CD8^+^ TILs were sensitized to tumor antigens, whereas the majority of them did not. Further investigations revealed that the differential CD39 expression was a key factor in discriminating the sensitivity of CD8^+^ TILs [153]. CD39 is a molecule implicated in chronic immune cell stimulation and is often markedly upregulated in a variety of malignant solid tumors. CD8^+^ CD39^+^ TILs are consistent with the characteristics of chronic antigen persisting stimulation, indicating that the activity of this class of TILs is suppressed. They retain the antitumor capacity but require additional stimuli to uncouple suppression, while ICB can then serve this function. Therefore, the higher the proportion of CD39^+^ in CD8^+^ TILs, the more likely that the PD-1/PD-L1 signaling axis will be effective. Hence, CD39^+^ is also a potential marker [154]. In addition, tumor microenvironment immune types (TMITs) were constructed and classified into four groups to describe different TMEs, based on PD-L1 expression and TILs. TILs are characterized by CD8A mRNA expression and the cytolytic activity score (CYT, GZMA, according mRNA expression levels of GZMA and PRF1). This stratification underscores the importance of PD-L1 expression and TIL recruitment. PD-L1-positive TILs are classified as TMIT type I. ICB therapy can benefit patients with PD-L1-positive TILs [155]. Moreover, in CRC, the number of mutations or neoantigens was significantly higher in TIMT type I (high PD-L1 and CD8A/CYT) than in TIMT type II (low PD-L1 and CD8A/CYT) cancers [155]. TMIT stratification may serve as a supplementary method to distinguish “cold” from “hot” tumors and to develop optimal immunotherapeutic strategies. The examination of TMIT may predict the therapeutic response of more diverse tumors to immune strategies on the basis of quantification of immune infiltration using mRNA-seq analysis.

### Polymerase epsilon (POLE) mutations

POLE is the key enzyme involved in DNA synthesis and repair processes, and the proofreading role of POLE is essential for replication fidelity, ensuring the appropriate replication of the genome during the cell cycle [156]. After POLE mutations, DNA repair defects and genetic material errors cannot be repaired. Over time, a large number of mutations have accumulated, even up to 10 times that of MSI-H CRC [157]. A retrospective analysis indicated that 66 (1.0%) of 6,517 CRC patients exhibited somatic POLE mutations. The patients with POLE mutations were relatively young, mostly male, and exhibited mainly lesions at the right side. They were also in the early stage of the disease (stage II–III) during initial diagnosis. Approximately 1–2% of MSS CRC exhibits a POLE mutation, while the frequency can range between 5 and 7% in patients aged < 50 years. Previous study has pointed out that CRC patients with POLE mutations are often accompanied with high levels of TILs, upregulated PD-L1 expression, and increased expression of cytotoxic T cell markers and effector cytokines, suggesting enhanced tumor immunogenicity [158]. POLE mutation seems to be a novel predictor of the response to ICB treatment. Wang et al. demonstrated that POLE mutations were independent biomarkers for determining the efficacy of immunotherapy across multiple cancer types [159]. Notably, MSS CRC patients with POLE mutations exhibit durable clinical responses from ICB therapy [23, 160]. This suggested that POLE mutations were a promising indicator that can be used to improve the benefits of immunotherapy on MSS mCRC patients [161].

### Neutrophil-to-lymphocyte ratio (NLR)

It is well established that tumor-related inflammation plays an important role in tumorigenesis, disease progression, and patient outcome. By contrast, systemic inflammation is associated with peripheral leukocyte alterations, which is manifested as alteration of the NLR. The NLR can predict the prognosis of patients with CRC and other solid tumors [162, 163]. Several retrospective studies have shown that high baseline NLR and an elevated NLR during treatment were significantly associated with poor outcomes, suggesting that NLR may be a potential predictive factor in patients who received ICB therapy [164–168]. In metastatic NSCLC, high NLR was associated with low response to immunotherapy and was an independent risk factor for poor prognosis [169]. Nevertheless, Jong et al. also indicated that the immune response was associated with a lower NLR at week 6 following immunotherapy, and a reduction in NLR during treatment was associated with longer PFS. As shown in previous studies from different centers, the specific baseline and the change range of NLR have not been previously demonstrated by large prospective studies. In addition, a limited number of studies have examined CRC patients. However, compared with other predictors, NLR is convenient for acquisition and monitoring, which is a research direction worth exploring.

Certain factors are closely associated with immune cell infiltration and the TME. However, their clinical value has not been confirmed and their importance is uncertain. We have compiled and listed these factors in Table 2. Importantly, the hyperprogression is a malignant phenomenon during ICB therapy. The related genes are sorted into Table 2.

**Table 2:** Other predictive markers of immunotherapy efficacy

*Positive Biomarkers*	
POLD1 mt	POLD1 gene encodes p125, the catalytic subunit of DNA polymerase δ. The polymerase activity and exonuclease function of DNA polymerase δ are concentrated in the p125 subunit, so the POLD1 gene is significantly involved in cell cycle regulation and DNA damage repair [156, 170]. CRC patients with POLD1 mutation often have the characteristics of microsatellite instability, suggesting that patients with POLD1 mutation may benefit from immunotherapy [159]
CDK12-Deficiency	CDK12 inactivation in prostate cancer is related to tandem genomic replication. CDK12 mutation may produce fusion related neoantigens and trigger an immune response, indicating that patients can benefit from ICB therapy [171, 172]
CDKN2A mt	CDKN2A is a tumor suppressor gene that induces cell cycle arrest in the G1 and G2 phases; it also suppresses the oncogenic action of CDK4/6 and MDM2 [173]. Tumors with JAK2 mutations or homozygous JAK2 deletions demonstrate allelic losses covering both the CDKN2A and JAK2 genes^.^ [174]
SERPINB3/4 mt	SERPINB3/4 mutations are able to enhance tumor neoantigen presentation. The results of a clinical study suggested that melanoma patients carrying SERPINB3/4 mutations could gain better benefit from treatment with CTLA4 antibodies [175]. In the clinical study with code number CA209-038, 68 patients with melanoma were enrolled who had progressed with or without prior to Ipilimumab therapy and received nivolumab. Five of six patients harboring SERPINB3/4 mutations had their disease controlled. However, due to the small sample size, no statistically significant associations between individual gene changes and treatment were found [176]
TP53/KRAS mt	Previous studies have found that in tumors with KRAS/TP53 mutation, the expression levels of PD-L1 and the infiltration of T cells were significantly increased. Patients with TP53, TP53/KRAS and KRAS mutations can benefit from PD-1 inhibitors [177, 178]
DNA DDR Genes mt	ATM, POLE, BRCA2, ERCC2/4, FANCA, CHEK1/2, MLH1/MSH2/MSH1, ATR, BAP1, and RAD belong to DDR genes, which have the function of DNA damage repair. Mutations in the DDR genes may increase the production of tumor neoantigens, resulting in higher tumor mutational burden [179, 180]
Fusobacterium nucleatum	Increased levels of Fusobacterium nucleatum were associated with improved treatment response to PD-L1 blockade [181]
CMTM6	A previous study suggested that CMTM6 expression in M2 macrophages may more accurately predict ICB response in CRC patients than the dMMR/MSI-H state. It can also identify pMMR CRC patients who may benefit from PD-1/PD-L1 inhibitors treatment [182]
NF1 mt	As a GTPase-activating protein, NF1 can downregulate RAS activity, and NF1 mutation can activate the MAPK signaling pathway [183]. In a previous study, patients with NF1 mutations, harboring high mutational burdens and high response rates, could benefit from anti–PD-1 therapy [184]
*Negative Biomarkers*	
MDM2/4	A previous study reported that MDM2/4 amplification could be used as an independent predictor of poor clinical outcome (time-to-treatment failure < 2 months) with immunotherapies. All six (4%) patients with MDM2/MDM4 amplifications indicated explosive progression of the disease. Notably, one of the patients exhibited a high TMB, which was considered as a responsive factor [185]
EGFR mt, ALK mt, MET rearrangement	Several clinical studies have shown that immunotherapy does not perform well in patients with driver gene mutations, such as EGFR and ALK, or patients with MET gene rearrangement, irrespective of the expression level of PD-L1 [186]
STK11 mt	STK11 is a tumor suppressor gene, and its mutations are related to the Peutz-Jeghers syndrome. Previous studies suggested that STK11 mutation can modify the “cold” TME, which was associated with decreased T cell infiltration, increased T cell exhaustion marker expression, and reduction in PD-L1 expression levels [187]. Moreover, the STK11 alteration was considered as a main driver of primary resistance to ICB therapy in KRAS-mutant lung adenocarcinoma samples [188, 189]; An additional study reported that the PFS and OS of the patients with KRAS/STK11 co-mutation who received ICB therapy were significantly lower than those in patients with KRAS mutation and STK11 wild-type patients. The concomitant present of KRAS and STK11 mutations was correlated with a greater risk of HPD following ICI monotherapy [190]
DNMT3A alteration	A previous study reported that in 155 patients, 4 of 5 patients harboring DNMT3A alteration had a hyperprogression (TTF < 2 months) with immunotherapies [185]
Loss of PTEN	PTEN loss was associated with reduction in T cell infiltration in the tumor samples, increased VEGF expression, and inferior outcomes with anti-PD-1 therapy [191]
DKK1	DKK1 inhibits antitumor immune activity of CD8+ T cells through the GSK3β/E2F1/T-bet axis. The increase in the serum expression of DKK1 can predict the poor tumor response to PD-1 blockade in dMMR/MSI CRC, whereas reversal of DKK1 neutralization may restore the sensitivity to PD-1 blockade [192]
LAGE3	High LAGE3 expression is associated with poor prognosis and poor immune infiltration in CRC patients, which suggests a poor immune response in ICB therapy [193]
Circulation LDH Levels	Previous studies have shown that baseline LDH alone or a combination of the LDH levels, performance status, and age were associated with response to ICIs in solid tumors. Another study indicated that LDH baseline levels were an independent indicator of PFS in melanoma patients treated with ICIs by Cox regression analysis [194–196]
Increased circulation Tsens	In a clinical study, the number of Tsens at baseline was detected, and patients with relatively high number of Tsens were prone to develop HPD. In contrast to those findings, the tumors subsided significantly in patients with lower Tsens. The results indicated that the number of Tsens in patients prior to immunotherapy could predict the risk of HPD. The baseline number of Tsens may represent the overall situation of a preexisting effector T cell with potential antitumor activity [197]
ILC3	ILC3 was specifically increased in HPD tumors [198]. Immunotherapy for Cancer ILC3s can respond to cytokine stimulation without specific antigen. ILC3 has been shown to produce IL-17 and IL-22, thereby promoting cancer progression [199]. This abnormal inflammatory environment may be related to the adverse efficacy of ICIS
T cell exhaustion	T cell exhaustion is defined as T cell dysfunction, decreased ability to recognize and eliminate antigens, and up regulation the expression levels of the inhibitory receptors, including PD-1, TIM3, TIGIT and LAG-3 [200]. Overexpression of these inhibitory receptors may be the key mechanism of PD-1 treatment resistance. Following the overexpression of these inhibitory receptors, CD8+ T cells indicates serious dysfunction in cytokine production, proliferation and migration [201]
Liver metastasis	Study demonstrated that melanoma patients with liver metastasis response worse than lung metastasis from ICB therapy [202]. MSS mCRC patients with liver metastasis also cannot benefit from the combination of TKI plus ICB [96, 203, 204]

*POLD1* DNA Polymerase Delta 1, Catalytic Subunit, DDR: Damage Response and Repair, *CDK12* Cyclin-Dependent Kinase 12, *SERPINB3/4* Serpin Family B Member 3/4, *CMTM6* CKLF like MARVEL Transmembrane Domain Containing 6, *STK11* Serine/Threonine Kinase 11, *ILC3* Group 3 Innate Lymphoid Cells, *Tsens* Senescent CD4 + T cells, *LDH* Lactate Dehydrogenase, *NF1* Neurofibromin 1, *MET* MET Proto-oncogene, Receptor Tyrosine Kinase, *DNMT3A* DNA Methyltransferase 3 Alpha, *DKK1* Dickkopf Wnt Signaling Pathway Inhibitor 1, *LAGE3* L Antigen Family Member, *3MET* MET Proto-oncogene, Receptor Tyrosine Kinase, *DNMT3A* DNA Methyltransferase 3 Alpha, *DKK1* Dickkopf Wnt Signaling Pathway Inhibitor 1, *LAGE3*: L Antigen Family Member 3

## The exploration of clinical strategies for ICB therapy and resistance in mCRC (summarized in Additional file 1: Table 1)

**Fig. 5:**
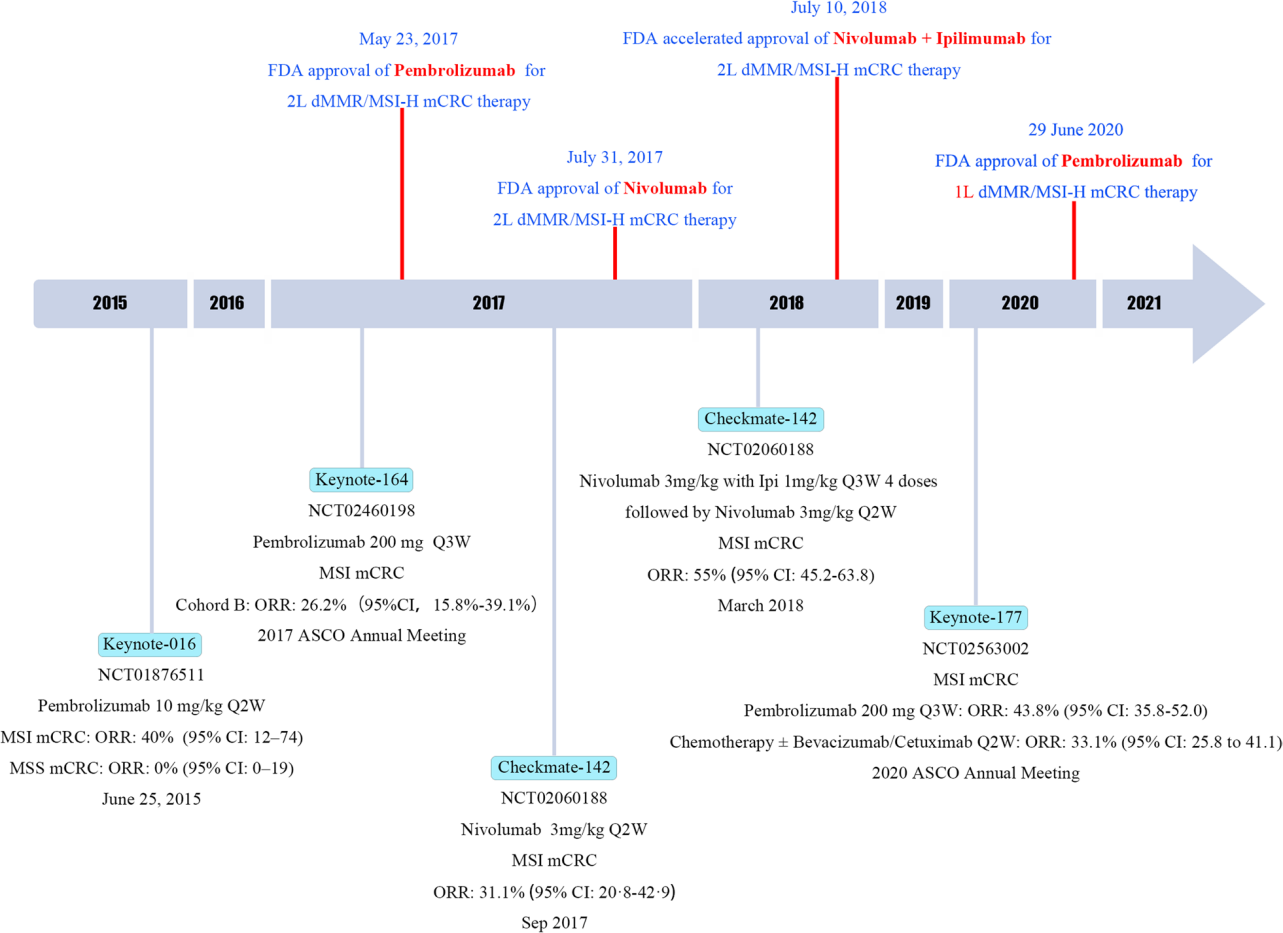
Timeline of ICB therapy in MSI-H/dMMR CRC

The important role of ICB therapy in MSI-H/dMMR CRC has received increasing attention, and certain drugs have been gradually approved and used in routine clinical practice (summarized in Fig. 5 and Table 3). However, 95% of mCRC patients are classified as MSS/pMMR. Therefore, the strategy to overcome immunotherapy resistance has been continuously explored. With regard to the immune microenvironment, MSS mCRC mostly belongs to the “immune-excluded tumor” and “immune desert tumor” classifications. The majority of the immune resistance mechanisms summarized above were noted in MSS mCRC, and the expression levels of cytotoxic cells, CD8^+^, Th1, Th2, follicular helper T cells, and T cell markers were significantly lower in MSS mCRC than those noted in MSI-H patients. Moreover, the TMB, percentage of missense or frameshift mutations, and the number of tumor neoepitopes were also significantly lower in MSI patients than those noted in MSI-H patients. The Keynote 016 study also demonstrated that MSS patients were largely refractory to immune monotherapy. However, the transformation of the “cold” MSS tumor into the “hot” tumor is an exploratory hotspot. Previous studies indicated that immunotherapy combined with MEK inhibitors or combined with anti-VEGF was not successful, whereas preliminary positive results have also been reported in MSS mCRC. (Selected results are summarized in Table 4.)

**Table 3:** Important clinical trials of immunotherapy in dMMR CRC

Clinical trial	NCT number	Phase	Status	Population	Arms and Interventions	Enrollment	ORR% (95% CI)	DCR% (95% CI)	PFS% (95% CI)	Significance
CheckMate-142	NCT02060188	2	Active, not recruiting	dMMR/MSI-H	[(Nivo 3 mg/kg + Ipi 1 mg/kg) Q3W] × 4 + (Nivo 3 mg/kg) Q2W^a^	119	55 (45.2–63.8)	12w: 79 (70.6–85.9)	9 m: 76 (67.0–87.2); 12 m: 71 (61.4–78.7)	FDA accelerated the approval of Nivo + Ipi for the second-line treatment of dMMR mCRC
					(Nivo 3 mg/kg) Q2W^a^	74	31.1 (20.8–42.9)	12w: 68.9 (57.1%–79.2)	12 m: 50.4(38.1%–61.4)	FDA-approved Nivo for the second-line treatment of dMMR mCRC
					(Nivo 3 mg/kg) Q2W + (Ipi 1 mg/kg) Q6W^b^	45	69% (53–82)	≥ 12w 84 (70.5–93.5)	12 m: 76.4 (60.5–86.6), 18 m: 76.4 (60.5–86.6), 24 m: 73.6 (57.2–84.5)	Exploration of immunotherapy in the first-line treatment of advanced MSI CRC
Keynote-164	NCT02460198	2	Completed	dMMR/MSI-H	Cohort A^e^	61	32.8 (21.3–46.0)^h^	50.8 (37.7–63.9)^h^	2.3 (2.1–8.1)^d^	Pemb was approved for MSI-H/dMMR mCRC patients after treatment progression with Fluorouracil, Oxaliplatin and Irinotecan
					Cohort B^e^	63	34.9 (23.3–48.0)^h^	57.1 (44.0–69.5)^h^	4.1 (2.1–18.9)^d^	
Keynote-016	NCT01876511	2	Completed	MSI	Cohort A: Pemb 10 mg/kg Q2W	41^ g^	28 m: 54 (37–69)^g^ 40 (12–74)^f^	28 m: 80 (65–91)^g^ 90 (55–100)^f^	20w: 88 (75–100) 28w: 70 (57–86)^g^	Pemb was approved for the treatment of all solid tumors carrying MSI-H/dMMR
				MSS	Cohort B: Pemb 10 mg/kg Q2W	25^ g^	28 m: 0 (0–14)^g^ 0 (0–19)^f^	28 m: 16 (5–36)^g^ 11 (1–35)^f^	20w:12 (4–36) 28w: 16 (6–41)^g^	
Keynote-177^i^	NCT02563002	3	Active, not recruiting	153 MSI	(Pemb 200 mg) Q3W	153	43.8% (35.8–52.0)	/	12 m: 55.3 (47.0–62.9) 24 m: 48.3 (39.9–56.2)	Pemb was approved by PDA for the first-line treatment of MSI mCRC
				153 MSI	Chemotherapy Q2W^c^	154	33.1% (25.8–41.1)	/	12 m: 37.3 (29.0–45.5) 24 m: 18.6 (12.1–26.3)	
NICHE	NCT03026140	2	Recruiting	dMMR	Ipi (1 mg/kg) on D 1 + Nivo (3 mg/kg) on D1 and D156	20	100% (86–100%)	/	/	Exploration of immunotherapy in neoadjuvant therapy
				pMMR	Ipi (1 mg/kg) on D 1 + Nivo (3 mg/kg) on D1 and D15 ± celecoxib from D1	15	26.7% (8–55%)	/	/	

*Nivo* Nivolumab, *Ipi* Ipilimumab, *Pemb* Pembrolizumab, *D* day, *w* Week, *m* Months

^a^Patients must have progressed on/after or been intolerant of at least one prior line of treatment, including a fluoropyrimidine and oxaliplatin or irinotecan; patients who refused chemotherapy were permitted on protocol

^b^Presented are results of nivolumab plus low-dose ipilimumab in the first-line therapy cohort from the phase II CheckMate-142 study

^c^Chemotherapy: 5-Fluorouracil based therapy ± Bevacizumab/Cetuximab

^d^Here showed the result of median PFS (95% CI)

^e^Cohort A: Participants were previously treated with at least one line of systemic standard of care therapy: fluoropyrimidine + oxaliplatin or fluoropyrimidine + irinotecan ± anti vascular endothelial growth factor (VEGF)/ epidermal growth factor regulator (EGFR) monoclonal antibody

Cohort B: Participants were previously treated with standard therapies, which must include fluoropyrimidine, oxaliplatin, and irinotecan

^f^The data are obtained from the article published on NEJM on June 25, 2015. At present, the final data have been updated

^g^The final results obtained from *ClinicalTrials.gov*

^h^Time Frame: Up to approximately 4 years

^i^Updated information: the cutoff date of the data was February 19, 2021

Pemb group: 36 m PFS1%: 42%; median-PFS1: 16.5 m (5.4–38.1); 12 m PFS2: 76%; 36 m PFS2: 60%; median-PFS2: 54.0 m (95% CI 44.4 m-NR) ORR: 45.1%

Chem group: 36 m PFS1%: 11%; median-PFS1: 8.2 m (6.1–10.2); 12 m PFS2: 67%; 36 m PFS2: 39%; median-PFS2: 24.9 m (95% CI 16.6–32.6) ORR: 33.1%

Pembro vs chemo met the prespecified criteria for PFS superiority at IA2. At final analysis, median PFS was 16.5 m vs 8.2 m (HR 0.59; 95% CI, 0.45–0.79), but did not meet statistical significance likely due to the high crossover rate from chemo to anti-PD1/PD-L1 therapies

**Table 4:** Important clinical trials of immunotherapy in MSS/pMMR mCRC

Combination Regimen	Clinical trial	NCT number	Phase	Status	Population	Arms and Interventions	Enrollment	ORR	DCR	PFS	Significance
Chemotherapy	KEYNOTE-651	NCT03374254	Ib	Active, not recruiting	MSS\pMMR mCRC	Cohort B: Pembrolizumab + mFOLFOX7	31	58.1%, 18 pts (1 CR, 17 PRs)	94%	\	\
						Cohort D: Pemb + FOLFIRI	31	15.6%, 5 pts (5 PRs)	63%	\	
	METIMMOX	NCT03388190	2	Recruiting	MSS\pMMR mCRC	Control Arm: FLOX	26	23.1% (6 pts)	\	m-PFS: 5.6 m(range, 0.5–15)	MSS mCRC patients may obtain ICB response through short-term oxaliplatin-based chemotherapy
						Experimental Arm: FLOX + Nivolumab	28	46.4% (13 pts)	\	m-PFS: 6.6 m (range, 0.5–20)	
Anti-EGFR	AVETUXIRI	NCT03608046	2	Recruiting	BRAF V600E wt, MSS mCRC, RAS wt	Two cohorts both received CET + IRI + AVE	59	60.0% (6\10)	\	6 m: 40.0% m-PFS: 4.2 m	This study reached the primary efficacy endpoint of RAS wt mCRC patients and brought dawn to the treatment of MSS mCRC
					BRAF V600E wt, MSS mCRC, RAS mt		59	61.5% (8\13)	\	6 m: 38.5% m-PFS: 3.8 m	
Anti-MEK	COTEZO IMblaze370	NCT02788279	3	Completed	Arm B: MSS\pMMR mCRC	Cobimetinib + Atezolizumab	183	2.7% (0.89–6.26) %	1.97% (1.77–3.81) %	m-PFS: 1.91 m (1.87–1.97)	To date, MSS mCRC can’t benefit from ICB combinations with MEK inhibitors
Anti-VEGF	BACCI	NCT02873195	2	Active, not recruiting	Arm A: 86.7% pMMR mCRC	Atezolizumab + Bevacizumab + Capecitabine	82	8.54% (3.5–16.8) %	\	m-PFS: 4.37 m (4.07–6.41)	The difference was not statistically significant in MSS patients
					Arm B: 85.7% pMMR mCRC	Placebo + Bevacizumab + Capecitabine	46	4.35% (0.53–14.84) %	\	m-PFS: 3.32 m (2.14–6.21)	
TKI	REGONIVO	NCT03406871	Ib	Completed	4% MSI 96% MSS	Regorafenib + Nivolumab	25	28% (7 pts)	\	m-PFS: 7.8 m (2.8- NR)	TKI in combination with ICB showed manageable safety profile and preliminary efficacy in unselected refractory MMS/pMMR/MSI-L mCRC patients; And liver metastasis appears to be a negative predictor of immunotherapy
	REGOTORI	NCT03946917	Ib\II	Recruiting	MSS\pMMR	Toripalimab + Regorafenib	33	15.2% (5.7–32.7) % Lung only100% (3\3) Liver only 0% (0\4) Liver and Lung 0% (0\14)	36.4% (21.0–54.9) %	m-PFS: 2.6 m (2.0–4.3)	
	LEAP-005	NCT03797326	2	Active, not recruiting	MSI-L\MSS\pMMR	Lenvatinib + Pembrolizumab	32	22% (9–40) %	47% (29–65) %	m-PFS: 2.3 m (2.0–5.2)	
Dual ICBs	LCCC1632	NCT03442569	2	Active, not recruiting	KRAS\NRAS\BRAF wt MSS mCRC	Ipilimumab + Nivolumab + Panitumumab	56	12w: 35% (21–48) %	\	m-PFS: 5.7 m (5.5–7.9)	\
	CCTG CO.26 Study	NCT02870920	2	Active, not recruiting	MSS\pMMR: 98.3%	Tremelimumab + Durvalumab + Best Supportive Care	119	0.8% (0.2–1.6) % (1 pt, MSI)	22.7%	m-PFS: 1.8 m (1.8–1.9)	\
					MSS\pMMR: 80.3%	Best Supportive Care	61	0 (0)	4.16% (1.40–12.3) %	m-PFS: 1.9 m (1.8–1.9)	
	MK-4280A	NCT02720068	1/2	Recruiting	MSS mCRC PD-L1 CPS ≥ 1	Favezelimab + Pembrolizumab	36	11% (3.1–26.1) %	\	m-PFS: 2.2 m (1.8–4.2) 6 m: 25.4%	Favezelimab in combination with Pembrolizumab showed manageable safety and antitumor efficacy in MMS/pMMR mCRC patients, Especially patients with PD-L1 CPS ≥ 1
					MSS mCRC PD-L1 CPS < 1		35	2.9% (0.1–14.9) %	\	m-PFS: 2.0 m (1.9–2.1) 6 m: 9.1%	
Anti-TGF-β	MP-VAC-204	NCT03724851	1b/2a	Active, not recruiting	MSS mCRC	Vactosertib 200 mg Bid + Pembrolizumab 200 mg Q3W	17	23.5%	\	1.3 m	Vactosertib seems to change the immune environment. Combined with ICB, it has a good antitumor effect in MSS mCRC patients
						Vactosertib 300 mg Bid + Pembrolizumab 200 mg Q3W	33	18.2	\	1.2 m	

### Immunotherapy combination with chemotherapy: issues that remain to be explored

The 2020 ESMO congress announced the progress of the Keynote-651 (NCT03374254) clinical trial, which is a multicenter, open-label, non-randomized phase Ib study aimed to assess the efficacy and toxicity of Pembrolizumab plus either mFOLFOX7 or FOLFIRI in mCRC [205, 206]. The results indicated an ORR of 58.1% in cohort B (Pembrolizumab plus mFOLFOX7) and an ORR of 15.6% in cohort D (Pembrolizumab plus FOLFIRI). Furthermore, a disease control rate (DCR) of 94% and 63% were noted, respectively. Pembrolizumab plus mFOLFOX7 or FOLFIRI demonstrated preliminary safety and efficacy in patients with MSS/pMMR mCRC. The METIMMOX study (NCT03388190) was reported at the ASCO congress, which compared the repeated sequential oxaliplatin-based chemotherapy (FLOX) combined Nivolumab versus FLOX alone as a first-line treatment of MSS mCRC. The results indicated that the mPFS of the FLOX plus Nivolumab group was 6.6 months (range, 0.5–20), whereas the ORR at 8 months reached 46.3%. This study suggested that FLOX therapy could convert MSS to an immunogenic state, allowing unresectable, previously untreated metastatic patients to achieve durable disease control following treatment with ICB [207]. The present study focused on the identification of predictive biomarkers of ICB responsiveness. Chemotherapy combined with immunotherapy has been the exploratory direction and the common method used by clinicians. Nevertheless, the aforementioned studies did not exhibit clear benefits, and additional investigations are required.

### Immune combination MEK inhibitors: changes to be made

Previous studies have shown that inhibition of MEK activity can induce a transcriptional signature similar to immune resistance in melanoma, suggesting that MEK inhibitor therapies may be cross-resistant to ICB therapies. Recently, Obenauf et al. demonstrated that dendritic cells (DCs), which are the key cells of the immune system, have lower activity and reduced cell number in a melanoma mouse model resistant to anti-MEK therapy. Moreover, stimulation of DCs restored the response to immunotherapy [208]. IMblaze370 (NCT02788279) is a multicenter, open-label, randomized controlled clinical trial exploring the efficacy of PD-L1 inhibitors in combination with a MEK inhibitor regimen (Atezolizumab plus Cabozantinib). Phase Ib results indicated a modest response rate (8%) and disease control (31%). However, the final results indicated that the combination therapy exhibited a median OS (mOS) of 8.87 months (95% CI 7.00 to 10.61) and a mPFS of 1.91 months (95% CI 1.87 to 1.97), which was not significantly different compared with other monotherapy groups. Additional adverse effects were also noted [209]. To date, several clinical trials have produced invalid results from ICB combinations with MEK inhibitors in CRC. However, certain preclinical studies have shown that a simple combination therapy is a suboptimal approach, while rational dosing and sequencing administration contributes to improved survival in mouse models [210]. The regimen of short-term ICB treatment prior to administration of MEK inhibitors is suggested to reverse drug resistance, which is more effective in prolonging tumor shrinkage and preventing the development of drug resistance. Certainly, relevant therapeutic approaches await exploration in high-quality clinical trials.

### Immunotherapy combined with anti-epidermal growth factor receptor (EGFR) therapy: Worth trying

The prognosis in all types of CMS4 tumor, with high APC and low BRAF mutation rates, is considerably poor [211]. The angiogenesis-associated pathway is aberrantly activated in this cancer type, promoting the hyperplasia and growth of metastatic tumor cells. The antiangiogenic drug Bevacizumab can significantly improve patient survival. A previous study indicated that Cetuximab exhibited a direct tumor killing effect, acting as an IgG1 monoclonal antibody that also exhibited antibody-dependent cell-mediated cytotoxicity (ADCC) effects. Cetuximab was also able to recruit anti-EGFR T cells as well as CD8^+^and CD3^+^ T cells. Moreover, this antibody can also increase PD-L1 expression and induce immunosuppression by possible synergism with ICB. The AVETUXIRI (NCT03608046) study investigated the combination of Bavencio with Cetuximab and Irinotecan in patients with refractory MSS mCRC who had failed prior standard therapy [212]. This study was stratified by the RAS mutation status into cohort A (RAS wild type) and cohort B (RAS mutated type). Initial findings indicated an ORR of 30% in cohort A, reaching its primary efficacy endpoint and proceeding to the phase II study. No PR was observed in cohort B, while both RAS wt and RAS mt groups exhibited DCRs of 60% (6/10) and 61.5% (8/13), respectively, mPFS of 4.2 and 3.8 months, and mOS of 12.7 and 14.0 months, respectively. The 6-month PFS rates were 40.0% and 38.5%, respectively. The 12-month OS rates were 53.3% and 57.7%, respectively. PR was not observed in the RAS mt cohort. However, optimal DCR, PFS, and OS data were also obtained in the RAS mt cohort, and the investigators established a RAS mt mCRC cohort with PFS as the primary endpoint in order to expand these research findings. In a single-arm, single-institution, phase I/II clinical trial (NCT04017650) [213], 26 patients with refractory MSS and BRAF^V600E^ metastatic CRC were recruited and Encorafenib, Cetuximab, and Nivolumab were used in combination. The ORR of 45% (95% CI 23 to 68) and DCR of 95% (95% CI 75% to 100), the mPFS of 7.3 months (95% CI 5.5 to NA) and the mOS of 11.4 months (95%CI 7.6 to NA) were reported. This study reached the predetermined efficacy endpoint, indicating that this novel regimen is effective and well tolerated in the treatment of MSS, BRAF^V600E^ mCRC.

### Immunotherapy combinations with tyrosine kinase inhibitors (TKIs) are anticipated to be scheduled in the future

Preclinical studies have shown that antiangiogenic therapy may improve the TME, increase and activate effector immune cells, reduce immunosuppressive cells, and relieve immunosuppressive effects, which play a significant role in the synergism of immunotherapy. However, the clinical effects of immunotherapy combined with anti-VEGF agents are not optimal. The BACCI (NCT02873195) study aimed to evaluate the efficacy of Capecitabine and Bevacizumab combined with Atezolizumab or placebo as the third-line treatment for refractory mCRC patients. The mPFS and mOS in the experimental group were 4.37 (95%CI 4.07 to 6.41), 10.55 (95%CI 8.21 to NA), and 3.32 (95%CI 2.14 to 6.21) and 10.61 (95%CI 8.80 to NA) in the control group [214]. The difference was not statistically significant in MSS patients, and the control group did not significantly improve OS. Inhibition of angiogenesis has long been considered as a potential approach to reverse immunotherapy resistance. However, immunization in combination with Bevacizumab has been shown to be unsuccessful. Despite these findings, immunotherapy combined with TKI has achieved promising results. TKIs, which also possess antiangiogenic effects, can block the three targets of VEGFR, notablyVEGFR3. Tyrosine kinases enzymes phosphorylate specific amino acids on substrate enzymes, which affect signal transduction pathway. TKIs exhibit a wide range of target inhibition effects, and it has been recognized that they may also inhibit colony-stimulating factor-1 receptor (CSF-1R). Based on their antiangiogenic effects, they can reverse antitumor immune activity by blocking CSF-1R-mediated pathway to inhibit tumor immune-related macrophages. Multiple prospective studies have been conducted worldwide to explore the efficacy of ICB with TKI therapy in the treatment of MSS mCRC.

The REGONIVO (NCT03406871) study in Japan was a phase Ib study using nivolumab plus regorafenib in refractory MSS CRC and gastric cancer. This study demonstrated an ORR of 28% and a mPFS of 7.8 months (95% CI, 2.8 to NR) in mCRC, with a 1-year PFS rate of 41.7% and a 1-year OS rate of 68.0%, which were considerably higher than the data from previous studies in MSS CRC [215]. However, this study could not be repeated in the follow-up North American REGONIVO phase II study (NCT04126733), and an ORR of 7.1%, mPFS of 8 weeks, and a mOS of 52 weeks were reported [204]. The REGOTORI (NCT03946917) study indicated that 5 out of 33 evaluable patients treated with 80 mg regorafenib achieved a tumor response with an ORR of 15.2% (95% CI, 5.7%–32.7%) and a DCR of 36.4% (95% CI, 21.0% to 54.9). The ORR was higher in patients without liver metastasis than that noted in those with liver metastasis (30.0% vs. 8.7%). The ORR of patients with only lung metastasis (3/3, 100%) was considerably higher than that of those with liver metastasis alone (0/4, 0%), suggesting that ICB combined with TKI is an option for refractory MSS mCRC, notably for patients without liver metastasis or lung metastasis alone [96]. A phase Ib study (NCT03903705) was performed to evaluate the safety and preliminary efficacy of Fruquintinib in combination with GB 226 for the treatment of mCRC. The data indicated favorable results. The ORR in 12 patients with MSS mCRC was 25.0%, whereas the DCR was 75%, and the mPFS was 5.45 months (95% CI 1.84 to 9.66) [216]. Recently, the largest trial of ICB combined with regorafenib in the treatment of MSS CRC (NCT03657641) was reported [217]. The subjects were patients with chemotherapy failure, of which 78% of patients had liver metastasis. The mPFS was 2.0 (95% CI 1.8 to 3.5) months and the mOS was 10.9 (95% CI 5.3 to NR) months. In 16 patients (23%) with non-hepatic metastatic disease, PFS was 4.3 (95% CI 1.9 to 8.4) months. Unfortunately, the trial did not reach its primary endpoint, and biomarker analysis is currently being conducted to further explore the benefit population.

### The efficacy of the dual immune checkpoint inhibitor combination therapy requires further validation

The CCTG CO.26 (NCT02870920) study evaluated the use of the dual ICB combination regimen as a post-line treatment for refractory mCRC. The experimental group received Durvalumab + Tremelimumab compared with the best supportive care (BSC) group, which was used as a control [218]. Notably, OS was significantly longer in the dual immunization group (6.6 months vs 4.1 months), whereas PFS was not prolonged (1.8 months vs. 1.9 months), and DCR was estimated to 22.6% and 6.6%, respectively. Subsequent analysis (excluding 2 patients with MSI-H) revealed that the median TMB was increased to 20.4, and the patients with a TMB > 28 MTs/MB could benefit more from dual immunotherapy, whereas high TMB in the BSC group was associated with a poor prognosis. The MEDITREME trial (NCT03202758) was a single-arm exploratory trial that enrolled 57 patients with RAS mutant, MSS, untreated mCRC, in which the investigators presumed that FOLFOX chemotherapy could induce immunogenic cell death and remove MDSCs, in order to potentiate the antitumor effects of immunotherapy. Enrolled patients received first-line therapy with FOLFOX in combination with Durvalumab and Tremelimumab, and the results indicated that the mPFS was not reached, with a 6-month PFS rate of 62.5% [219]. Another multicenter phase II study LCCC1632 (NCT03442569) met the primary study endpoint of a remission rate of 35% at 12 weeks [220]. The study was designed to evaluate the efficacy and safety of Ipilimumab and Nivolumab plus Panitumumab in patients with KRAS/NRAS/BRAF wild-type MSS mCRC. A total of 49 patients were evaluated with regard to the efficacy of treatment at 12 weeks, 34.7% of the patients (17/49) exhibited PR, 0 had CR, and 42.9% (21/49) exhibited stable disease (SD). The mPFS was 5.7 months (95% CI, 5.5–7.9 months). This chemotherapy-free immune combination targeted therapy regimen offers first-line hope for the chemotherapy-resistant advanced RAS/BRAF wild-type MSS mCRC patients. Of course, phase III studies are still required for confirmation. As an important new immune checkpoint, LAG-3 is structurally similar to CD4. It has four extracellular regions that bind to ligands, thus inducing immune cell failure and reducing cytokine secretion. An increase in reliable clinical data on the double blocking of LAG-3 and PD-1 has prompted people to focus on this combination immunotherapy. A clinical trial (NCT02720068) [221] demonstrated that the combination of LAG-3 inhibitor (Favezelimab) and Pembrolizumab exhibits good antitumor activity, especially in patients with PD-L1 CPS ≥ 1.

## Future perspectives

Immune checkpoint inhibitor therapy is relatively less toxic than chemotherapy and targeted therapy. However, some of its unique adverse effects reduce its clinical efficacy, and certain rare adverse effects could be life-threatening, which result in skin, endocrine, hepatic, gastrointestinal, pulmonary, and skeletal muscle toxicity. The related aspects of this topic will not be covered in this review.

ICBs have been successfully used in the MSI-H CRC population. Pembrolizumab, Nivolumab, and Ipilimumab have been approved to be used in MSI-H refractory mCRC, and Pembrolizumab has been recommended as first-line therapy of treatment. More recently, clinical trials indicated that neoadjuvant immunotherapy may have the potential to become the standard therapy for CRC patients. In a stepwise exploration, ICBs hold promise as adjuvant therapies for patients with stage III CRC after resection. Moreover, the combination with specific drugs is expected to improve efficacy and attenuate associated toxicity. The urgent task is to find multiple biomarkers and formulate standardized scoring standards in order to screen population and benefit more patients. For the MSS population, which constitutes the majority of CRC patients, ICB monotherapy was ineffective. In addition to the microsatellite status, other potential biomarkers can be developed that can aid the identification of potential populations. More importantly, it is significant to develop measures that can turn “cold tumor” into “hot tumor” so as to expand the application scope of immunotherapy. This scope of research may provide a milestone in CRC treatment.

## Supplementary Information

**Additional file 1**. Summary of Clinical Trials in This Paper.

## Data Availability

The datasets used and analyzed during the current study are available within the manuscript and its additional files.

## Abbreviations

mCRC: Metastatic colorectal cancer
ICB: Immune checkpoint blockades
MSI-H: Microsatellite instability—high
mOS: Median overall survival
NSCLC: Non-small cell lung cancer
PD-1: Programmed death-1
PD-L1: Programmed cell death-ligand 1
FDA: Food and Drug Administration
dMMR: Deficient mismatch repair
pMMR: Mismatch repair-proficient
TMB: Tumor mutation burden
TME: Tumor microenvironment
5-FU: 5-Fluorouracil
TAAs: Tumor-associated antigens
TCB: T cell bispecific antibody
TSAs: Tumor-specific antigens
MHC-I: Major histocompatibility complex class I
TAPs: Transporter associated with antigen processing
β2M: β-2-Microglobulin
TCF: T cell factor
NK cell: Natural killer cell
STAT3: Signal transducer and activator of transcription 3
MAPK: Mitogen-activated protein kinase
VEGF: Vascular endothelial growth factor
IL-8: Interleukin 8
PTEN: Phosphatase and tensin homologue
PI3K: Phosphatidylinositol 3-kinase
Tregs: Regulatory T cells
TAMs: Tumor-associated macrophages
MDSCs: Myeloid-derived suppressor cells
IFNGR: IFN-γ receptor
JAK1/JAK2: Janus kinase 1/Janus kinase 2
IRF1: Interferon regulatory factor 1
PTPN2: Protein tyrosine phosphatase non-receptor type 2
NTCs: Non-tumor cells
IPRES: Innate PD-1 resistance
MEK: Mitogen-activated protein kinase kinase
PFS: Progression-free survival
FMT: Fecal microbiota transplantation
PR: Partial response
CR: Complete response
CMS: Consensus molecular classification
TGF- β: Transforming growth factor β
Th17: T helper 17
CIRC: Coordinate immune response cluster
MSI-H: Microsatellite high instability
MSI-L: Microsatellite low instability
MSS: Microsatellite stability
TMB-H: TMB high
muts/MB: Mutations/megabase
mPFS: Median progression-free survival
TNB: Tumor neoantigen burden
CPS: Combined positive score
APCs: Antigen-presenting cells
ISGS: IFN-stimulated genes
ISGS. RS: ISGS resistance signature-related genes
TILs: Tumor-infiltrating lymphocytes
TMITs: Tumor microenvironment immune types
POLE: Polymerase epsilon
DCR: Disease control rate
DCs: Dendritic cells
EGFR: Epidermal growth factor receptor
ADCC: Antibody-dependent cell-mediated cytotoxicity
TKIs: Tyrosine kinase inhibitors
CSF-1R: Colony-stimulating factor 1 receptor
BSC: Best supportive care
SD: Stable disease
