# Supplementary material for: Exploring immunotherapy in colorectal cancer
Source: J Hematol Oncol. 2022 Jul 16;15:95. doi: 10.1186/s13045-022-01294-4 (PMC9288068; doi:10.1186/s13045-022-01294-4)
Supplement: Supplementary file 1 — Additional file 1. Summary of Clinical Trials in This Paper. [file 13045_2022_1294_MOESM1_ESM.pdf]

Supplementary Table 1. Summary of Clinical Trials in This Paper

Strategy	NCT Number	Status	Study Results	Interventions	Primary Endpoint	Phases	Enrollment	Start Date	Completion Date
Vaccines	NCT04912765	Recruiting	No Results Available	Biological: Neoantigen Dendritic Cell Vaccine|Drug: Nivolumab	24-mo RFS; IIRAV	2	60	April 15, 2021	May 2025
	NCT03287427	Not yet recruiting	No Results Available	Drug: TetMYB Vaccine|Drug: BGB-A317	≥Grade 3 AEs Rates	1	32	May 30, 2018	December 30, 2022
	NCT04117087	Recruiting	No Results Available	Drug: KRAS peptide vaccine|Drug: Nivolumab|Drug: Ipilimumab	Drug-Related Toxicities Numbers; FcIPMKSTCs	1	30	May 29, 2020	June 1, 2024
	NCT04046445	Recruiting	No Results Available	Drug: ATP128|Drug: BI 754091|Drug: VSV-GP128	AEs; SAEs; PFS	1| 2	96	July 22, 2019	December 31, 2023
	NCT03639714	Active, not recruiting	No Results Available	Biological: GRT-C901|Biological: GRT-R902|Biological: nivolumab|Biological: ipilimumab	AEs; SAEs; ORR; RP2D	1| 2	214	February 13, 2019	March 2023
Chemotherapy	NCT03388190	Recruiting	No Results Available	Drug: Nivolumab|Drug: FLOX	PFS	2	100	May 29, 2018	December 31, 2021
	NCT03374254	Active, not recruiting	No Results Available	Biological: Pembrolizumab|Drug: Binimetinib|Drug: Oxaliplatin|Drug: Leucovorin|Drug: 5-Fluorouracil [5-FU] |Drug: Irinotecan	DLT%	1	220	February 16, 2018	November 16, 2023
	NCT03202758	Unknown status	No Results Available	Drug: Durvalumab, Tremelimumab and, FOLFOX	Safety	1| 2	48	August 29, 2017	August 29, 2020

	NCT04895137	Recruiting	No Results Available	Drug: mFOLFOX6+Bevacizumab+PD-1 monoclonal antibody treatment combinations	PCR rate	2	42	May 1, 2021	May 1, 2024
	NCT04301557	Recruiting	No Results Available	Drug: PD-1 Antibody|Drug: Oxaliplatin|Drug: Capecitabine|Radiation: External beam radiotherapy|Procedure: Total mesorectal excision	pCR rate	2	25	July 31, 2020	December 30, 2024
Antigen Modulation	NCT05061017	Recruiting	No Results Available	Drug: Pixatimod|Drug: Nivolumab|Drug: Cyclophosphamide (low dose)	ORR	2	61	October 28, 2021	September 2026
COX-Inhibitor	NCT03926338	Recruiting	No Results Available	Drug: Neoadjuvant therapy with PD-1 inhibitor plus COX inhibitor|Drug: Neoadjuvant therapy with PD-1 inhibitor	pCR rate	1| 2	34	May 10, 2019	May 1, 2022
	NCT03638297	Recruiting	No Results Available	Drug: PD-1 antibody + cox inhibitor	Response rate	2	27	August 16, 2018	August 31, 2021
Neoadjuvant Therapy	NCT03854799	Recruiting	No Results Available	Drug: Avelumab|Drug: Capecitabine|Radiation: EXTERNAL---BEAM IRRADIATION 50.4 GY	pCR rate	2	101	April 1, 2019	December 2023
Wnt-Inhibitor	NCT03447470	Recruiting	No Results Available	Drug: RXC004|Drug: Nivolumab	DLT	1	50	March 18, 2019	December 2021
	NCT02521844	No Results Available	No Results Available	Drug: ETC-1922159|Drug: Pembrolizumab	MTD; RD; AEs; ABDIAs; AbECG; CECOGPSs; AbCLTs; AbVSMs; FGRSEs	1	83	October 2015	April 2023

PD-L1-Inhibitor	NCT03428126	Active, not recruiting	No Results Available	Drug: Durvalumab|Drug: Trametinib	MTD; BOR (CR+PR)	2	29	March 21, 2018	November 2021
TGF-β-Inhibitor	NCT02947165	Completed	No Results Available	Drug: NIS793|Drug: PDR001	DLTs, AEs, SAEs	1	120	April 25, 2017	June 18, 2021
	NCT03724851	Active, not recruiting	No Results Available	Drug: TEW-7197	MTD	1| 2	67	November 28, 2018	August 1, 2023
MAPK-Inhibitor	NCT02788279	Completed	Has Results	Drug: Atezolizumab (MPDL3280A), an Engineered Anti-PDL1 Antibody|Drug: Cobimetinib|Drug: Regorafenib	OS	3	363	July 5, 2016	December 26, 2018
VEGF-Inhibitor	NCT02873195	Active, not recruiting	Has Results	Drug: Atezolizumab|Biological: Bevacizumab|Drug: Capecitabine|Other: Laboratory Biomarker Analysis|Other: Placebo	PFS	2	133	July 7, 2017	March 27, 2023
	NCT04715633	Recruiting	No Results Available	Drug: PD-1 inhibitor plus VEGF inhibitors	cCR; pCR	2	52	December 1, 2020	December 31, 2022
LAG3-Inhibitor	NCT02720068	Recruiting	No Results Available	Biological: Favezelimab|Biological: Pembrolizumab|Drug: Oxaliplatin|Drug: Irinotecan|Drug: Leucovorin (Calcium Folinate) |Drug: Fluorouracil [5-FU] |Biological: Favezelimab/Pembrolizumab|Drug: Lenvatinib	DLT; AEs	1	576	May 2, 2016	December 13, 2023
	NCT03642067	Recruiting	No Results Available	Drug: Nivolumab|Drug: Relatlimab	ORR	2	96	February 12, 2019	February 2024
Dual-Drug	NCT03442569	Active, not recruiting	Has Results	Drug: Panitumumab|Drug: Nivolumab|Drug: Ipilimumab	ORR	2	56	March 9, 2018	March 31, 2024

	NCT04258111	Active, not recruiting	No Results Available	Biological: IBI310 (anti-CTLA-4 antibody) |Biological: Sintilimab(anti-PD-1 antibody)	ORR	2	4	August 27, 2020	May 2023
	NCT02870920	Active, not recruiting	Has Results	Drug: Tremelimumab<br>Drug: Durvalumab<br>Other: Best Supportive Care	OS; PFS; ORR	2	180	August 10, 2016	December 31, 2021
Demethylation	NCT02959437	Terminated	Has Results	Drug: Azacitidine|Drug: Pembrolizumab|Drug: Epacadostat|Drug: INCB057643|Drug: INCB059872	AEs; ORR	1| 2	70	February 27, 2017	March 2, 2020
	NCT02811497	Completed	No Results Available	Drug: Azacitidine|Drug: Durvalumab	ORR	2	28	September 2016	August 4, 2020
Deacetylation	NCT02890069	Active, not recruiting	No Results Available	Biological: PDR001|Drug: LCL161|Drug: Everolimus|Drug: Panobinostat|Drug: QBM076|Drug: HDM201	DLTs; FDIR; AEs; SAEs; CBPLP; Dose intensities	1	298	October 14, 2016	February 3, 2022
	NCT02512172	Active, not recruiting	No Results Available	Drug: Oral CC - 486|Drug: Romidepsin|Drug: MK - 3475	TIL; DLT	1	27	February 19, 2016	January 2022
TKI	NCT04963283	Recruiting	No Results Available	Drug: Cabozantinib|Drug: Nivolumab	DCR	2	46	June 23, 2021	February 9, 2025
	NCT04866862	Not yet recruiting	No Results Available	Drug: Combination of Fruquintinib and Camrelizumab	ORR	2	32	May 1, 2021	April 30, 2024
	NCT04819516	Recruiting	No Results Available	Procedure: high-intensity focused ultrasound therapy|Drug: Toripalimab	AEs; AbCLTs	1	10	December 7, 2020	December 2025
	NCT04764006	Recruiting	No Results Available	Drug: Surufatinib	ORR	2	10	October 13, 2021	November 1, 2023

	NCT04110093	Recruiting	No Results Available	Drug: Regorafenib and PD-1 inhibitor	ORR; PFS	1| 2	120	March 1, 2019	August 31, 2021
	NCT03797326	Active, not recruiting	No Results Available	Biological: Pembrolizumab|Drug: Lenvatinib	ORR; AEs	2	590	February 12, 2019	December 22, 2023
	NCT03977090	Recruiting	No Results Available	Drug: Geptanolimab Injection|Drug: Fruquintinib	AEs; DLT; RDE	1	21	April 4, 2019	December 2021
	NCT03946917	Active, not recruiting	No Results Available	Drug: JS001|Drug: regorafenib tablet	MTD; DLT; ORR	1| 2	44	March 12, 2019	November 20, 2021
	NCT03903705	Recruiting	No Results Available	Drug: Fruquintinib in Combination with Sintilimab	DLT; MTD; AEs; ORR; DCR; PFS; OS	1| 2	208	April 25, 2019	July 2021
Radiotherapy	NCT04001101	Recruiting	No Results Available	Combination Product: RT and Anti-PD-1|Drug: Anti-PD-1	ORR	2	140	October 10, 2019	April 2024
PI3K-Inhibitor	NCT03711058	Recruiting	No Results Available	Drug: Copanlisib|Drug: Nivolumab	MTD; ORR	1| 2	54	January 17, 2019	January 2022
STAT3-Inhibitor	NCT03647839	Completed	No Results Available	Drug: Nivolumab 10 MG/ML|Drug: BNC 105|Drug: BBI608	ORR	2	90	September 6, 2018	April 9, 2021
Triple-Drug	NCT05035381	Recruiting	No Results Available	Drug: Pembrolizumab (PD-1 Inhibitor) Combined with Bevacizumab and FOLFIRI Regimen	ORR	2	10	January 1, 2019	December 30, 2022
	NCT05019534	Recruiting	No Results Available	Drug: Vemurafenib Oral Tablet [Zelboraf]|Drug: Cetuximab Injection [Erbitux]|Drug: Camrelizumab	RP2D; MTD	1	12	May 1, 2021	December 1, 2021

	NCT04940546	Recruiting	No Results Available	Drug: Sintilimab|Drug: Oxaliplatin|Drug: Capecitabine|Drug: Bevacizumab	AEs	1| 2	30	June 16, 2021	June 30, 2023
	NCT04547166	Not yet recruiting	No Results Available	Drug: HLX10/Placebo|Drug: HLX04/Avastin	OS; PFS; ORR; DOR; DCR	3	666	September 18, 2020	June 30, 2025
	NCT04194359	Recruiting	No Results Available	Drug: sintilimab injection|Drug: Control Test	PFS	3	436	February 4, 2021	June 2025
	NCT04072198	Recruiting	No Results Available	Drug: Nivolumab|Drug: Bevacizumab|Drug: Irinotecan|Drug: Oxaliplatin|Drug: Leucovorin|Drug: fluoruracil	ORR	2	70	September 26, 2019	September 2022
	NCT03608046	Recruiting	No Results Available	Drug: Avelumab|Drug: Cetuximab Injection|Drug: Irinotecan	ORR	2	59	October 3, 2018	December 31, 2023
	NCT03414983	Active, not recruiting	No Results Available	Biological: Nivolumab|Drug: Oxaliplatin|Drug: Leucovorin|Drug: Fluorouracil|Drug: Bevacizumab	PFS	2| 3	195	February 20, 2018	June 30, 2023
Arginase-Inhibitor	NCT02903914	Active, not recruiting	Has Results	Drug: INCB001158|Drug: Pembrolizumab	AEs	1| 2	260	September 14, 2016	November 8, 2021
FMT	NCT04729322	Recruiting	No Results Available	Procedure: Biopsy|Procedure: Fecal Microbiota Transplantation|Drug: Fecal Microbiota Transplantation Capsule|Drug: Metronidazole|Drug: Neomycin|Biological: Nivolumab|Biological: Pembrolizumab|Other: Questionnaire Administration|Drug: Vancomycin	ORR	Early Phase 1	15	February 22, 2021	December 31, 2021
TCB	NCT02650713	Completed	No Results Available	Drug: Atezolizumab|Drug: RO6958688		1	228	January 7, 2016	January 13, 2020

TKI: Tyrosine Kinase Inhibitor; FMT: Fecal microbiota transplantation; 24-mo RFS: 24-month Relapse Free Survival ;IIRAV: Induced immune response against vaccinated NAs; AEs: adverse events; FcIPMKSTCs: Fold change in interferon-producing mutant-KRAS-specific CD8 and CD4 T cells at 16 weeks; SAEs: serious adverse events; PFS: Progression-free survival; ORR: Objective Response Rate; RP2D: recommended

Phase 2 dose; DLT: dose limiting toxicity; pCR: Pathological Complete Response; MTD: Maximum Tolerated Dose; RD: Recommended Dose; ABDIAs: adverse bone density imaging assessments; AbECG: abnormal 12-lead electrocardiogram (ECG) readings; CECOGPSs: Change in Eastern Cooperative Oncology Group performance status; AbCLTs: abnormal clinical laboratory test results; AbVSMs: abnormal vital sign measurements; FGRSEs: fatigue and gastrointestinal-related side effects; BOR: Best Overall Response; OS: Overall Survival; cCR: Clinical complete response; DCR: Disease Control Rate; RDE: Extended period recommended dose; DOR: Duration of response; FDIR: Frequency of dose interruptions and reductions; CBPLP: Changes between baseline and post-baseline laboratory parameters and vital signs; TIL: Degree of change in tumor infiltrating lymphocytes.
